# Cross-species gene modules emerge from a systems biology approach to osteoarthritis

**DOI:** 10.1038/s41540-017-0014-3

**Published:** 2017-05-17

**Authors:** Alan James Mueller, Elizabeth G. Canty-Laird, Peter D. Clegg, Simon R. Tew

**Affiliations:** 1 Department of Musculoskeletal Biology, Institute of Ageing and Chronic Disease, Faculty of Health and Life Sciences, University of Liverpool, William Henry Duncan Building, 6 West Derby Street, Liverpool, L7 8TX UK; 2 The MRC-Arthritis Research UK, Centre for Integrated Research into Musculoskeletal Ageing (CIMA), Liverpool, UK

## Abstract

Complexities in degenerative disorders, such as osteoarthritis, arise from multiscale biological, environmental, and temporal perturbations. Animal models serve to provide controlled representations of the natural history of degenerative disorders, but in themselves represent an additional layer of complexity. Comparing transcriptomic networks arising from gene co-expression data across species can facilitate an understanding of the preservation of functional gene modules and establish associations with disease phenotypes. This study demonstrates the preservation of osteoarthritis-associated gene modules, described by immune system and system development processes, across human and rat studies. Class prediction analysis establishes a minimal gene signature, including the expression of the Rho GDP dissociation inhibitor *ARHGDIB*, which consistently defined healthy human cartilage from osteoarthritic cartilage in an independent data set. The age of human clinical samples remains a strong confounder in defining the underlying gene regulatory mechanisms in osteoarthritis; however, defining preserved gene models across species may facilitate standardization of animal models of osteoarthritis to better represent human disease and control for ageing phenomena.

## Joint degeneration: linking animal and human disease

Cartilage, and other tissues in our joints, begins to degenerate with age resulting in pain and reduced mobility; this is termed osteoarthritis (OA). To understand OA better researchers have often used animal models to represent this disease; however, these models have never been fully-evaluated against human cartilage. This study considered the messages produced by cartilage cells in both humans and rats. Using a method that creates a network of messages the study was able to define “blocks” of cell messages that were associated with diseased cartilage in both the rat and human. As part of this study the authors also defined a set of messages that could be used to distinguish healthy and disease cartilage. In this way it may be possible to define patients with early OA that may benefit from therapeutic interventions. (135)

## Introduction

Disorders of cartilage and joints account for a high incidence of disability^1^ and are prevalent co-morbidities of the ageing population.^2^ Debilitating in their own right musculoskeletal disorders contributes significantly to the global burden of disease, being the fourth most prevalent disorder^3^ and have a wider impact on the rehabilitation of co-occurring pathologies (obesity, stroke, and cardiovascular disease), thereby representing a major health policy issue.

Osteoarthritis (OA), considered a chronic, degenerative condition of multiple tissues that comprise a joint,^4^ results in the destruction of cartilage, the friction-free interface, leading to considerable functional impairment. The major cell population of cartilage, chondrocytes, account for the unique extracellular matrix (ECM), which confers compression resistance and gliding characteristics of normal cartilage.^5^ Despite considerable efforts to characterize the nature of degenerate cartilage the pathophysiological process is not fully understood, disease-associated genetic variants are limited, and there are no disease-modifying therapeutics available.^6^

Disease complexity, arising from multiscale perturbations, makes a mechanistic understanding of OA difficult. OA is a complex disease because it involves multiple tissues, environmental factors, behaviors, signaling pathways and genes. For example, numerous genetic risk loci, epigenetic effects, inflammation associated with ageing^7^ and obesity^8^ and biomechanical factors contribute to joint degeneration. Although heritable factors account for 50% of an individual’s risk of developing OA, only 16 disease risk loci have been consistently identified^9^ with candidate genes such as *GDF5* and *SMAD3* harboring the most promising risk alleles;^10^ overall, multiple risk alleles are likely to contribute to OA susceptibility. Additionally, OA is dynamic, being progressive and chronic, and so is likely to involve the dysregulation of several biological systems over multiple timescales. As with other multifactorial diseases (e.g., neurological disorders), analysis of individual components cannot adequately explain the properties of the whole system (the contributing tissues) as novel properties emerge with increasing complexity of the system.^11^

Animal models of multifactorial disorders are used to provide a controlled representation of subsets of human disease and aim to reproduce the natural history and progression. Rodent models of cartilage pathophysiology are frequently employed and include surgical-induced (destabilization of the medial meniscus) and chemical-induced (monoiodoacetate joint injection) OA. The rat is frequently used in the study of OA; however, there is no single standardized in vivo model.^12^ Gene expression studies arising from these models are often poorly controlled, underpowered, combine joint tissues, and use multiple different gene expression analysis platforms, making comparison across studies problematic. Overall, animal models that better represent human OA are required.^13^

Weighted gene co-expression network analysis^14^ is a systems biology methodology that considers the connectivity between genes based upon their co-expression. This method facilitates investigation of the global network properties of a transcriptome and provides functional insights into the organization of a co-expression network by utilizing the concept of scale-free networks.^15^ As the co-expression of genes encodes the downstream protein interactions, the study of transcriptional co-expression patterns can reveal emergent functional properties of the cellular system under investigation. Weighted gene co-expression network analysis (WGCNA) has been used widely to define candidate genes for human disorders including prognostic signatures for cancers, and has demonstrated preservation of functional gene modules between human and mouse brains.^16^ In this context network, nodes represent genes that are expressed in a sample. Edges connect nodes based upon their weighted co-expression across samples. WGCNA assumes that all nodes are connected and the connections have different strengths; highly connected genes within a network can be gathered as modules, with “hubs” being the most highly connected genes within a module. The modularity of networks is inherent to cell biology,^17^ and biological phenomena arise from molecular interactions organized into functional modules. The network topology (or architecture of these module structures) can be compared across networks to assess conservation of modules in different conditions or between species.

The system under consideration in this study was the chondrocyte, either as whole cartilage or isolated cells. Transcriptomic profiling from different environments and conditions provided information on perturbations to that system. The study sought to establish, from publically available gene expression data, a comprehensive analysis of the gene–gene co-expression networks from transcriptomic profiles of different chondrocyte phenotypes in human and rat. By performing this analysis on human and rat data, an understanding of the preservation of network module topology would inform the validity of rodent in vivo models of OA. Additionally, by establishing a subnetwork of genes associated with the phenotype of interest, osteoarthritic cartilage, rational therapeutic and diagnostic targets may be proposed for future study. This study demonstrates high preservation of modules across species associated with physiological functions in addition to modules associated with inflammatory mediators and system development that are characteristic of a subset of human osteoarthritic cartilage samples. Importantly, genes with class discrimination potential were established that may serve to define early cartilage degeneration.

## Results

### Construction of rat and human co-expression networks

**Fig. 1:**
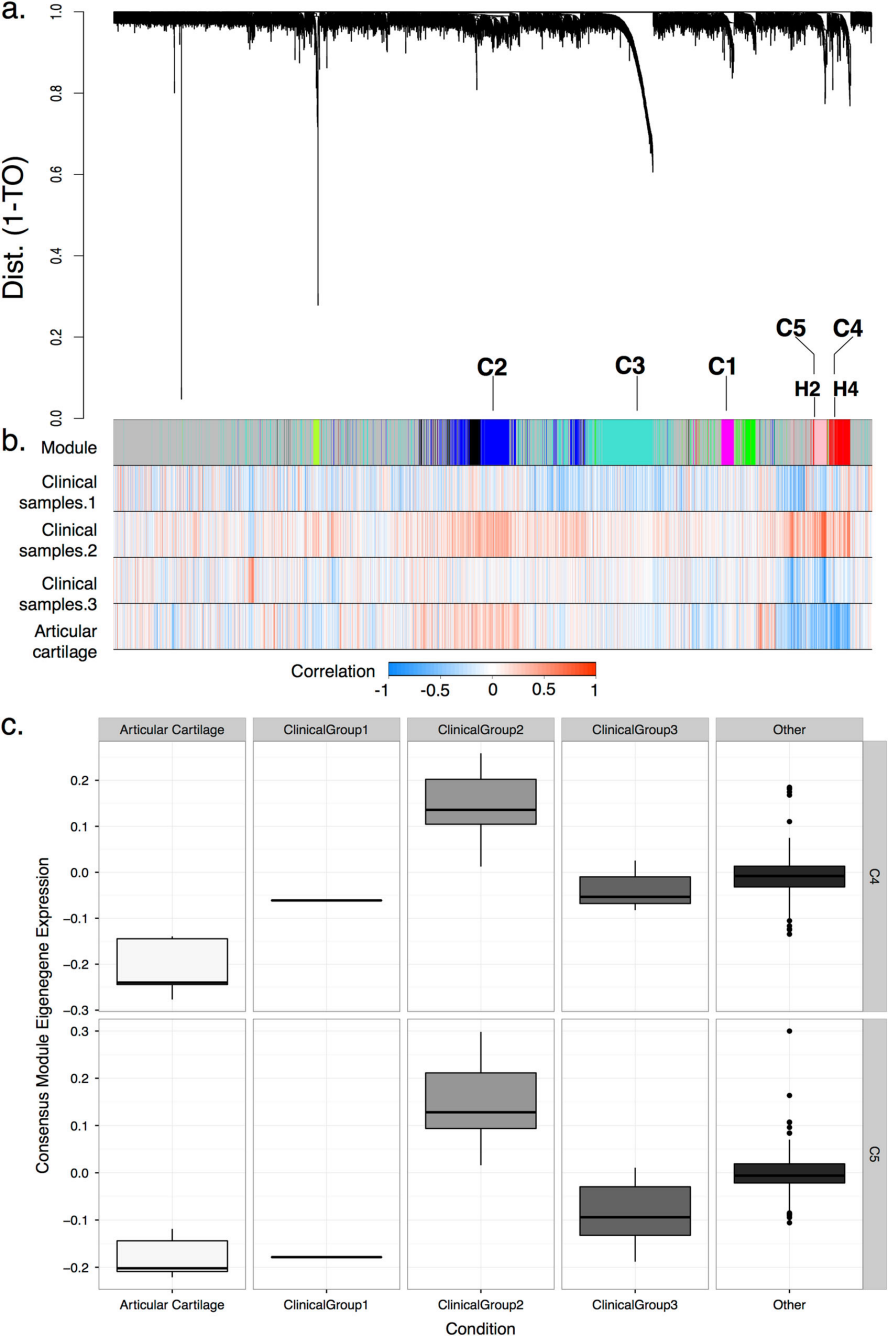
Definition of co-expression modules in the human (**a**). Hierarchical cluster dendrogram derived from merged human gene expression data (derived from *n* = 129 arrays and 5982 genes) defines 12 modules. *Branches* of the dendrogram represent groups of genes. Dynamic tree-cutting was used to define modules; where these had significant overlap, they were assigned the same label (arbitrary module color). The co-expression distance (1-topological overlap (TO)) between the genes is defined by the *y*-axis; the genes are plotted along the *x*-axis; **b** *top band*—gene modules (clusters of highly co-expressed genes) coded by *color*; unassigned genes are colored “*gray*”. Key modules of interest (H2 and H4) are annotated; the associated consensus modules (C1 to C5), modules found in both rat and human networks, are also defined above the module bar. Alphanumeric module codes are provided in Supplementary Fig. 4a; *bands 2–4*—selected samples showing positive or negative correlations with genes enriched in each module (see figure key—*red* corresponds to positive correlation). Clinical samples represent whole-cartilage gene expression derived from human donors; gene expression profiles from samples were allocated to one of three clinical sample groups (“Clinical Groups 1–3”); a fourth (“Articular cartilage”) represents ostensibly normal cartilage. The overlap between human and consensus modules is provided in Supplementary Fig. 4b. “Clinical Group 2” shows a positive correlation with H2 and H4 modules, which overlap with the C4 and C5 consensus modules; **c** module eigengene expression (*y*-axis) is defined for two consensus modules (C4 and C5) across selected samples (*x*-axis) relative to all samples contributing to the human network (“Other”). For both consensus modules there is a significant difference in the expression of the corresponding module eigengenes across samples using a non-parametric Kruskal–Wallis one-way analysis of variance (C4, *brown*—*p* = 1.6e−09; C5, *yellow*—*p* = 2.7e−11) with high expression found in “Clinical Group 2” relative to normal articular cartilage. Overall, whole-cartilage samples demonstrate heterogenous gene expression and differ in their association with network modules

**Fig. 2:**
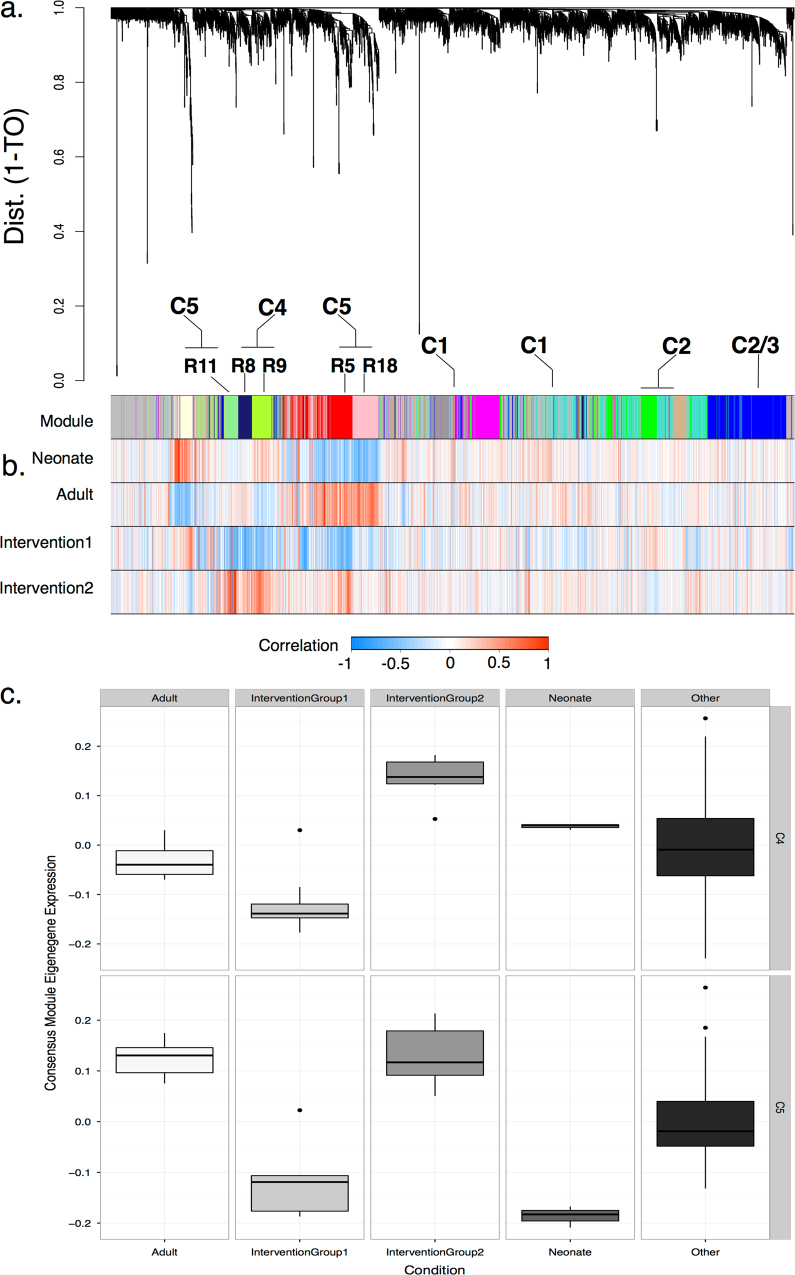
Definition of co-expression modules in the rat. **a** Hierarchical cluster dendrograms in the rat, derived from gene expression data (*n* = 115 arrays and 5982 genes) show 20 modules. An alphanumeric code is provided to clarify references to specific modules (Supplementary Fig. 5a). Unassigned genes are colored *gray*; **b** *top bar*—modules are colored below the dendrogram and represent highly co-expressed genes. Key modules of interest (R5, R8, R9, R11, R18) are annotated. Some consensus modules are split across two or more rat network modules. Consensus modules, modules of co-expressed genes that are shared by two or more co-expression networks, are presented above the module bar; consensus module colors and codes are comparable between species and can be related to those in Fig. 1b and Supplementary Fig. 5b; *bands 2–4*—selected trait groups showing positive or negative correlation with genes enriched in each module (see figure key—*red* corresponds to positive correlation). Complete module–trait associations are provided in Supplementary Fig. 5a. Whole-cartilage samples derived from neonate and adult rat cartilage show reciprocal correlations with some modules. Whole-cartilage samples derived from in vivo intervention studies (“Intervention Group 1–2”) also show reciprocal correlations with rat modules that contribute to the C4 and C5 consensus modules; **c** Module eigengene expression (*y*-axis) is defined for two consensus modules (C4 and C5, *right-hand vertical bars*) across selected samples (*x*-axis, “Condition”) relative to all other samples (“Other”). Across sample groups both module eigengenes, using a non-parametric Kruskal–Wallis chi-square test, are found to be significantly differentially expressed with high expression found in “Intervention Group 2” relative to other selected traits (C4—*p* = 4e−05; C5—*p* = 2.8e−08) including “Intervention Group 1” and “Adult” rat cartilage. Overall, subsets of rat whole-cartilage samples derived from in vivo joint intervention studies demonstrate heterogenous gene expression and differ in their association with network modules

Global co-expression networks were constructed from rat (115 arrays) and human (129 arrays) gene expression data using 5982 genes with common annotation by WGCNA. Overall, 12 modules were defined for the human network (Fig. 1a, b) and 20 modules for the rat (Fig. 2a, b), inclusive of a module of unassigned genes for each species. All further characterizations were undertaken on these modules. An alphanumeric code for each module (H-human, R-rat) is provided for reference, Supplementary Figs. 4a and 5a. The genes assigned to each module are listed Supplementary Data SD1 and SD2.

### Consensus modules established between species

To establish concordance and divergence in the network organization across the two species, consensus modules, modules shared by both networks, were derived from each species network using the weighted average of the two correlation matrices. In all, there were six consensus modules (C1–C6), Figs. 1b and 2b and Supplementary Figs. 4b and 5b. Functional annotation of the consensus modules included: “muscle system process” (C1), “oxidative phosphorylation” (C2), “cell cycle” (C3), “system development” (C4), and “immune system process” (C5). Unassigned genes were placed in a single module (C6, colored “gray”). See Supplementary Data SD36–41 for full annotations and gene lists.

### Module–trait relationships define subsets of whole-cartilage samples in both species

To understand the possible functions of these modules and their relevance to chondrocyte dysregulation, the association between each module eigengene (ME) and traits of interest were established. Traits groups were defined as samples derived from similar experimental conditions (e.g., monolayer expansion of chondrocytes), anatomical sites (e.g., growth plate zones), and phenotypic groups (e.g., different ages); MEs were representative genes for each module. Whole-cartilage clinical samples for human were obtained at surgery (humans, age range: pre-adolescent–82 years old) and defined as healthy or osteoarthritic; rat whole cartilage was obtained following an in vivo surgical intervention in rats (e.g., joint destabilization model of OA) or from normal tissue post mortem. In both species, gene expression profiles of whole cartilage did not show strong statistical associations with network modules based upon the published phenotypic groups (e.g., healthy or osteoarthritic cartilage). Whole-cartilage samples were assigned to new groups (“Clinical Group 1–3”, humans, or “Intervention Group 1–2”, rats) based upon co-clustering of gene expression profiles by multidimensional scaling. These new groups demonstrated associations with species-specific modules (Supplementary Figs. 4a and 5a).

### Functional annotation of trait-associated modules

**Fig. 3:**
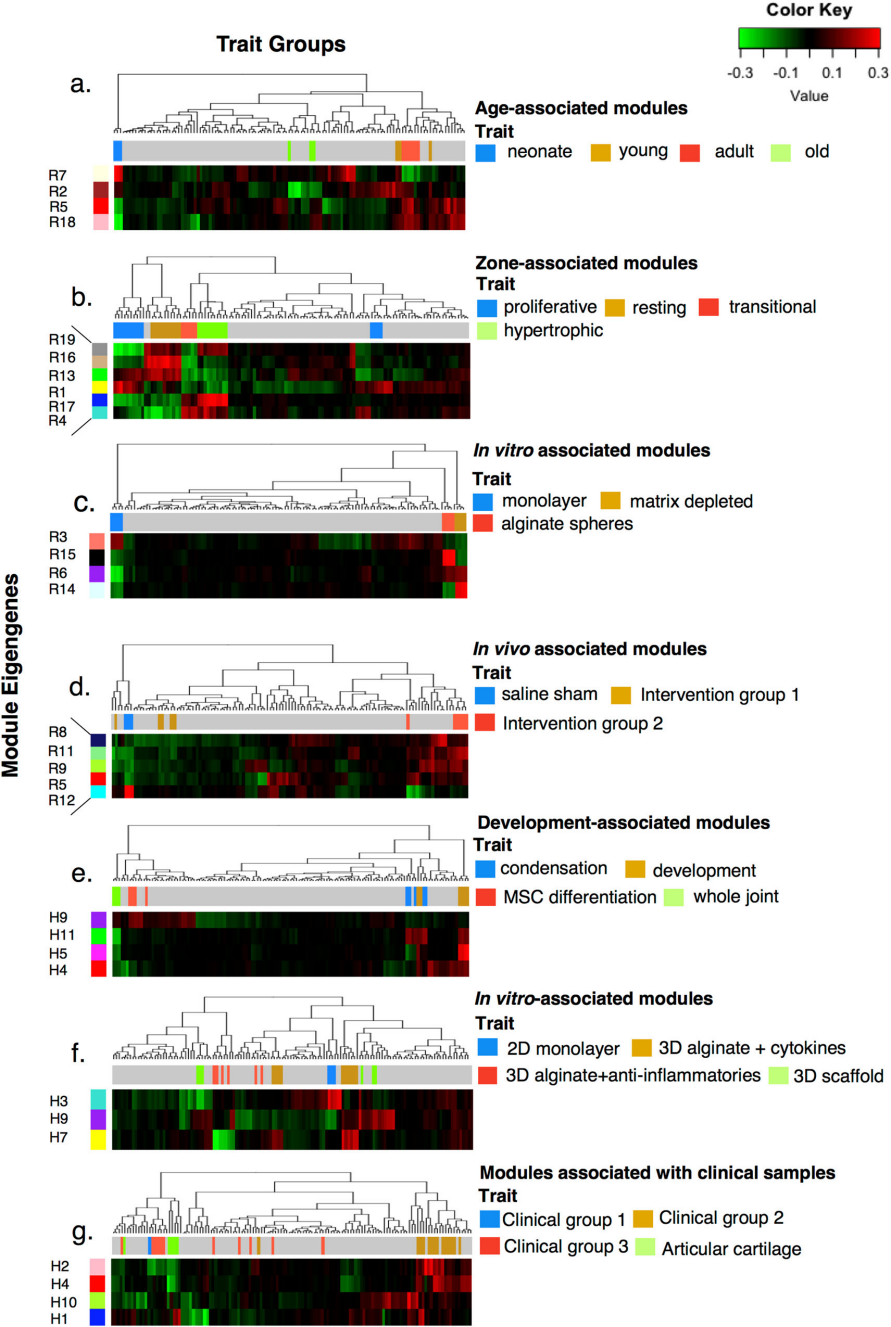
Module eigengene heatmaps for associated traits. Module eigengenes (*left-side vertical bars*, rows; R—rat, H—human) derived from network modules with strong trait associations are presented as heatmaps of module eigengene expression. Supporting module–trait correlations and results of statistical tests are presented in Supplementary Figs. 4a (human) and 5a (rat). Samples associated with traits of interest are *colored* above the heatmap (*top bar*, *columns*) according to the associated key; samples with no strong module association are colored *gray*. Dendrograms indicate sample clusters. Module eigengene expression is indicated as high (*red*) or low (*green*), see color key. **a**–**d** Rat module eigengenes show distinct expression signatures for several trait groups (age, growth plate zones, in vitro chondrocyte studies, and in vivo joint interventions). Patterns of expression differ between cartilage samples derived from different age groups (**a**) and between different in vivo intervention groups (**d**); **e-g** equivalent heatmaps are presented for human trait groups (development and differentiation studies, in vitro studies including both two-dimensional and three-dimensional chondrocyte cultures, and whole cartilage clinical samples). The difference in expression profile between the clinical subgroups and normal articular cartilage may be visualized in (**g**). Gene ontology functional annotations for all modules are provided in Supplementary Data SD3–32

The unique ME expression profiles for trait groups are presented in Fig. 3 **a–d** for rat and in Figs. 3 **e–g** for human. Age-associated rat modules (Fig. 3a) were significantly enriched with functional annotations for RNA metabolic process, immune system process, and muscle system process. MEs associated with in vivo interventions (Fig. 3d) had annotations for immune system process, skeletal system development, sterol biosynthetic process, and cell adhesion. Functional annotations for in vitro studies and growth plate zone-associated modules (Fig. 3b, c) are found in Supplementary Table 1 and Supplementary Data SD3–SD21. Human chondrocyte condensation and differentiation profiles (Fig. 3e) were associated with modules annotated for multicellular organismal development, cell differentiation, and muscle system process. Whole-cartilage samples from clinical samples (Fig. 3g) divided into groups associated with modules annotated for positive regulation of the immune system and cell differentiation (“Clinical Group 2”) or with oxidative phosphorylation (normal articular cartilage). Further functional annotations for human modules are found in Supplementary Table 2 and Supplementary Data SD22–SD32.

### Subset of human clinical samples associated with immune system and differentiation modules

Further consideration was given to trait associations between subsets of whole-cartilage samples showing the greatest separation from other cartilage samples (“Clinical Group 2”) and the H2 and H4 modules (immune system process and cell differentiation annotations, respectively). These modules were negatively correlated with normal articular cartilage samples (Fig. 1b and Supplementary Fig. 4a). The H2 and H4 modules demonstrated the greatest overlap with the C4 and C5 consensus modules (Supplementary Fig. 4b). Differential expression analysis of the eigengenes for these modules with clinical sample groups found a significant difference across all groups with the greatest difference in expression found between “Clinical Group 2” and other whole-cartilage subsets (Fig. 1c).

### Rat module–trait relationships reveal distinct associations with age and growth plate zones

In vivo joint interventions in rats could not be readily discriminated into experimental groups (e.g. surgical sham or joint destabilization surgery) and were classified into interventional groups based on gene expression clustering (“Intervention Groups 1–3”). A group consisting of predominantly surgical interventions (“Intervention Group 2”), associated with the R5, R8, R9, and R11 modules, were annotated for “system development,” “response to wounding” and “immune system process”. These were found to be comparable to the C4 and C5 modules (Fig. 1b and Supplementary Fig. 5b). Sham control samples (isotonic saline joint injections) and “Intervention Group 1” were strongly associated with the R12 module containing genes associated with “skeletal system development” and “cartilage development”. Differential expression of C4 and C5 MEs in rat whole-cartilage samples was significantly different across groups with a subset (“Intervention Group 2”) showing higher expression (Fig. 2c). Similar to human whole-cartilage sample, subsets of rat cartilage had positive associations with the C4 and C5 modules.

Age-associated modules were also defined from the rat network (Fig. 3a and Supplementary Fig. 5a). Neonatal cartilage samples were negatively correlated with R5 and R18 modules, while adult and early-aged cartilage samples demonstrated the inverse relationship. In this case both cartilage from older rats and cartilage from “Intervention Group 2” were associated with the R5 module. The R2 module had a moderate association (cor = 0.35, *p* = 2e−04) with aged rats, but no association with intervention studies (Supplementary Fig. 5a). Absolute ages were not available in public data sets.

### Cross-species module preservation statistical analysis

To establish how well the modules defined in the larger reference set (rat, 20 modules) were preserved and reproducible in the test network (human, 12 modules), module preservation statistics were calculated for each reference-test module pair using a series of permutation tests for measures of module density and connectivity. Thirteen rat modules were shown to have well-defined human counterparts (summary *Z*-score>5), while some appeared specific to the rat network (Supplementary Table 1 and Supplementary Data SD35). Modules associated with physiological processes (RNA metabolic process, cell cycle, immune system process, and skeletal system development) were strongly preserved between species (summary *Z*-score > 10). The module of unassigned genes (R20) also demonstrated high preservation; however, this contained the majority of the genes (Supplementary Fig. 5b). A “toy” module of 100 randomly assigned genes collected from all possible genes (R21, “gold”; Supplementary Table 1) demonstrated no evidence of preservation across species.

### Differential eigengene network analysis shows strong preservation of network structure across species

**Fig. 4:**
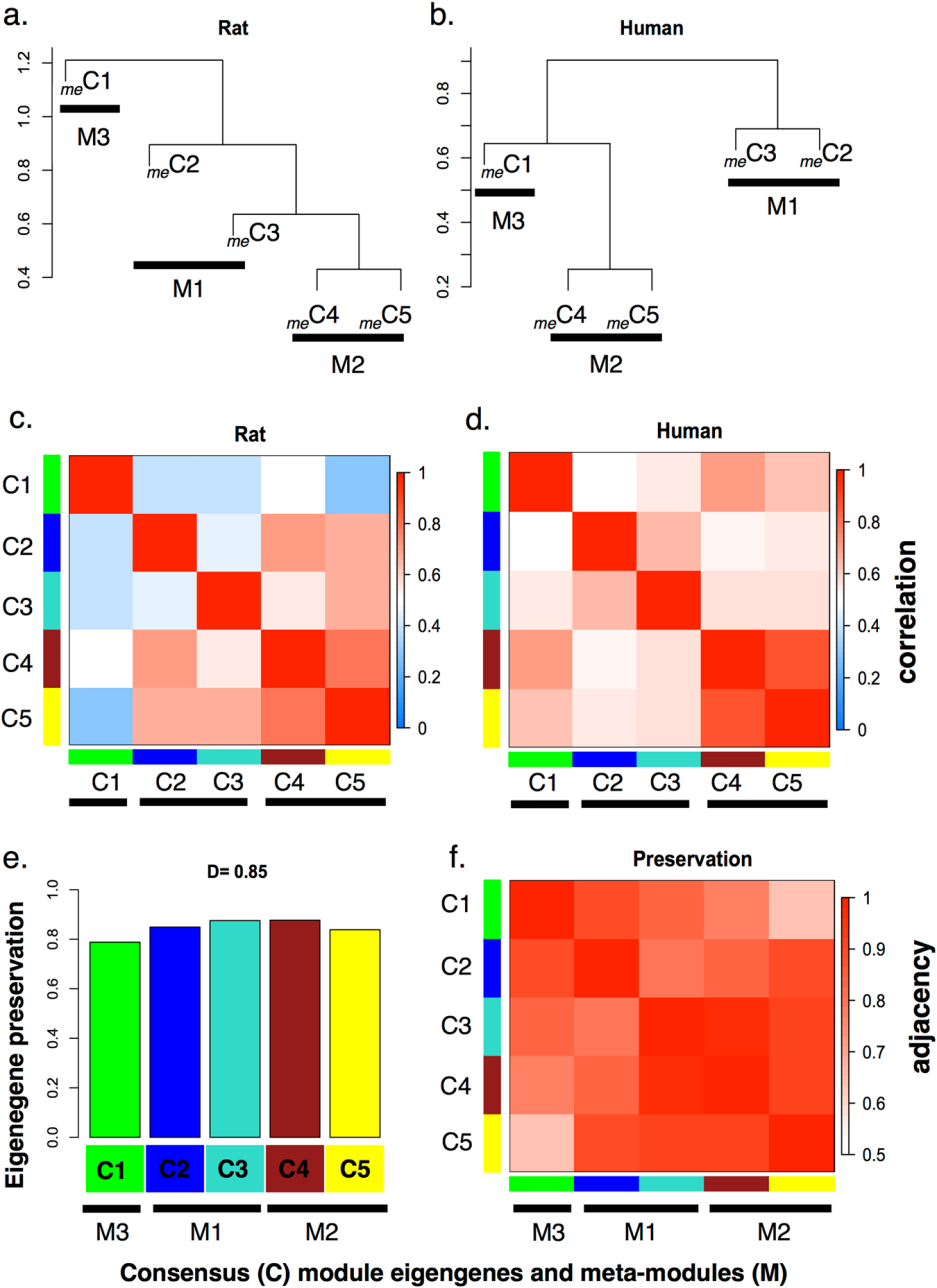
Differential consensus module eigengene analysis. Differential eigengene network analysis between rat and human consensus module eigengene networks was used to define the strength of the correlation preservation for all eigengene pairs across the two networks: **a**, **b** Clustering dendrograms of consensus module eigengenes (*me*) demonstrates the presence of meta-modules M1–M3 (*thick horizontal bars*) across both species; the same three major branches are found in both *dendrograms*. **c**, **d** Heatmap plots of eigengene adjacencies for each of the eigengene networks (**c**, rat; **d**, human). Each of the *rows* and *columns* indicate an eigengene labeled by the consensus module color. *Red* indicates high adjacency (positive correlation), while *blue* indicates the inverse, as depicted by the color legend. **e** *Bar plot* of the preservation of the consensus eigengene relationships between the two networks. Each *bar* represents the eigengene of the associated consensus module; the eigengene preservation measure is given by the height of the *bar* (*y*-axis). The high-density value *D*(Preserv^human,rat^) = 0.85 reflects the high overall preservation between the two networks, i.e., correlation preservation is high between all pairs of eigengenes across the two networks. **f** Adjacency heatmaps for the pair-wise preservation network Preserv^human,rat^; high values of Preserv^human,rat^ indicate correlation preservation between pairs of module eigengenes is strong across the two networks. Each consensus module eigengene is represented by the *rows* and *columns*, with the level of *red* saturation indicating adjacency according to the color legend. Overall, the M2 meta-module in both species, derived from C4 and C5 eigengenes, are the most highly correlated. The C3 and C4 modules represented the most highly preserved eigengenes

Differential eigengene network analysis (Fig. 4a–f) was used to define the overall preservation of the correlation of consensus ME pairs across the two species networks. To assess the overall preservation of modules and connectivity across the two data sets, eigengene networks were prepared based upon correlations between each pair of consensus MEs. This analysis sets out to establish whether consensus modules C4 and C5, associated with whole-cartilage subsets in both the rat and human, were conserved in the global network structure. There was strong evidence for eigengene network preservation between rat and human (density, *D*(Preserv^human,rat^) = 0.85). Consensus MEs from the human data were defined by three main groups, or meta-modules (Fig. 4b). The first (M1) consisted of the C2 and C3 modules (blue and turquoise), the second (M2) of the C4 and C5 modules (yellow and brown), and the third (M3) contained the C1 module (green). This configuration was approximated in the rat data, particularly the preservation of the M2 meta-module (Fig. 4a). This demonstrated that in addition to the C4 and C5 modules being (i) present in both species, (ii) associated with gene expression profiles of subsets of whole cartilage, (iii) the organization of these functional units was also preserved across the networks and were highly correlated in their expression in cartilage from two species.

### Meta-module M2 associated with cell differentiation and immune system related to distinct cartilage samples

Overall, the M2 meta-module represented 165 genes differentially expressed in subsets of cartilage samples from both rat and human, thus representing a biologically relevant superset of genes. Collectively, these genes were significantly enriched (after false discovery rate correction) for functional annotations including: “immune system process”—*CTSC*, *CCR1*, *MMP9* (*p* = 3.1e−16), “response to wounding”—*TLR7*, *GAP43* (*p* = 1.8e–8), “cell adhesion”—*TNN*, *CAM1*, *COL18A1* (*p* = 1.5e−5), and “system development”—*SOX17*, *SOX18*, *DMP1*, *IL18* (*p* = 3.7e−6). The M2 meta-module was significantly enriched for evidence of protein–protein interactions using STRING (*p* = 0). These modules were also enriched for distinct canonical signaling pathways: C4—“PI3K-Akt signaling pathway” (*q* = 2.9e−4) and “ECM interaction” (*q* = 3.2e−5); C5—“immune system” (*q* = 1.3e−11), and “osteoclast differentiation” (*q* = 3.4e−7) using over-representation analysis.

### Systems development module C4 associated with osteophytic and hypertrophic samples across species

The structure and the nature of the most highly connected genes in the M2 modules were considered further. The C4 consensus module was associated with the H4 human module (Supplementary Fig. 4b), but are split across the R8 and R9 modules in the rat (Supplementary Fig. 5b). There was low to moderate correlation of module membership *k*ME values between the rat and human modules (R8: cor = 0.27, *p* = 4.7e−3; R9: cor = 0.39, *p* = 3.2e−07) indicating some preservation of hub modules. Consensus hub genes for the C4 module included *CXCL12*, *CTSK*, *DMP1*, *ACP5*, MMP9, and *COL1A1*; however angiogenesis-associated genes *EMCN* (endomucin) and *KDR* (kinase insert domain receptor) were both found to be the most highly connected hubs in both species (Supplementary Fig. 6a, b). A subset of human OA cartilage samples (“Clinical Group 2”, cor = 0.28, *p* = 1e−3), osteophytic cartilage, and developing chondrocyte samples were associated with the H4 module; in the rat data the equivalent modules (R8 and R9) were associated with hypertrophic chondrocytes (R8, cor = 0.37, *p* = 4e−05) and a subset of OA intervention models, “Intervention Group 2” (R9, cor = 0.37, *p* = 6e−05). In summary, rat and human modules associated with the C4 module were also associated with subgroups of cartilage samples associated with degenerate whole cartilage, osteophytic cartilage, or hypertrophic chondrocytes.

### Consensus module C5 reveals conserved network of immune system genes in cartilage

**Fig. 5:**
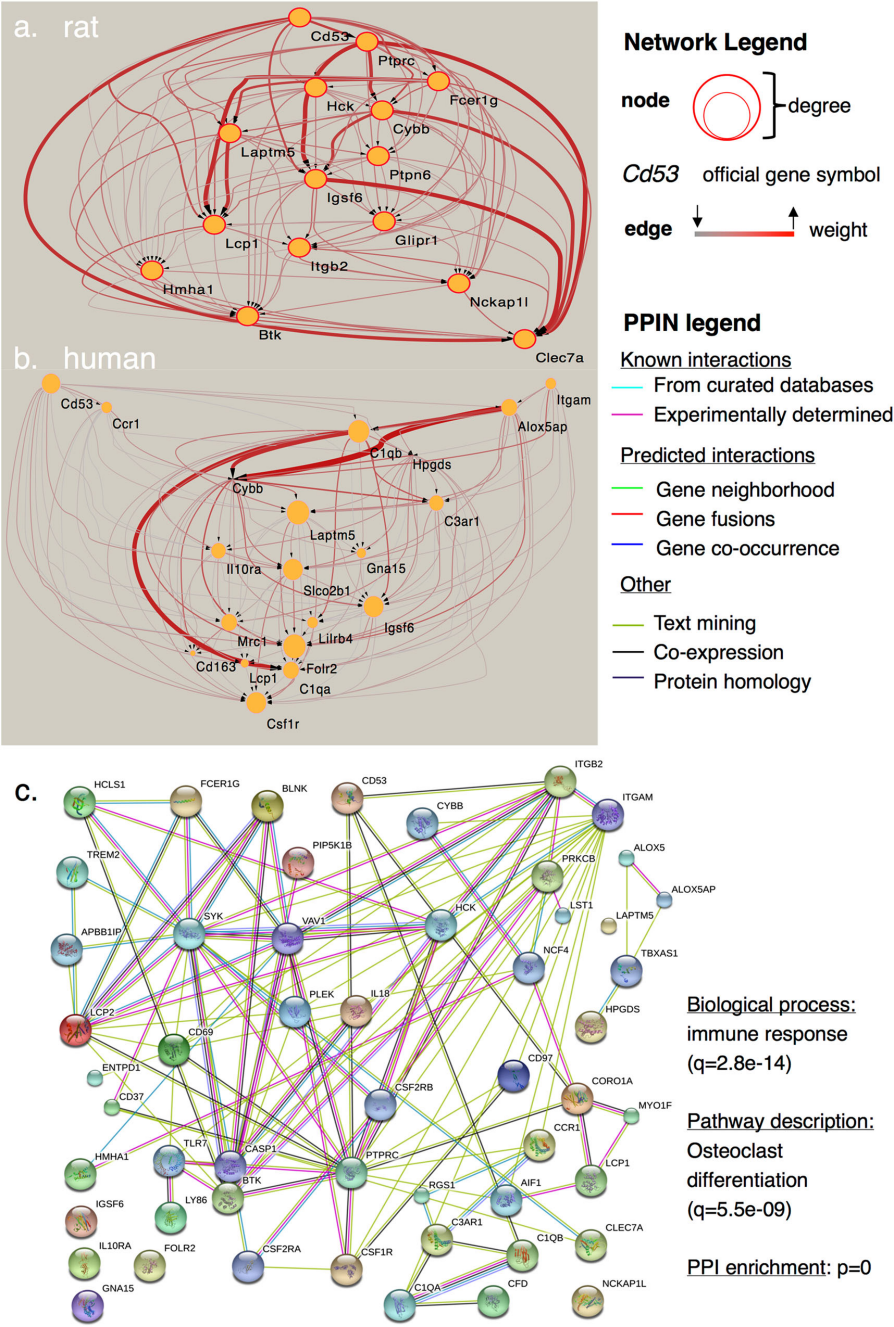
Visualization of module subnetworks. **a** Rat R5 module corresponds with the C5 consensus module and is annotated with the term “inflammatory system process”. Highly interconnected nodes (genes) included *Nckap1l*, *Btk*, *Cd53*. **b** Human H2 module also corresponds with the C5 consensus module and is associated with a subset of clinical cartilage samples (“Clinical Group 2”). Multiple highly connected genes are shared between the two modules including *CD53*, *CYBB*, *LCP1*, *IGFS6*. Other highly connected hub genes (module membership, *k*ME > 0.6) including *Alox5ap* (rat, *k*ME = 0.81) and *NCKAP1L* (human, *k*ME = 0.71) were also found in the modules from the other species, but did not pass filtering thresholds for network representation. In general the equivalent nodes in the human network show more sparse connectivity as described by the size of the node (network legend), for example, *LCP1*. Key describes increasing node degree (number of connections) by increasing node diameter; edges (connecting nodes) vary with color and thickness to indicate the weighted co-expression between nodes. Highly connected nodes are larger and have multiple thick and red edges. **c** Protein–protein interaction network, PPIN (STRING evidence view, PPIN legend) derived from the C5 consensus module genes demonstrates that gene co-expression networks can represent functional networks highly enriched for protein–protein interactions. The most significant biological process and pathway descriptors are provided

The C5 module described both the rat R5 and R11 and human H2 and H4 modules and was highly conserved. In the rat the R5 module was positively associated with cartilage samples derived from adult/early-aged rats and a subset of intervention studies modelling OA (“Intervention Group 2”). In the human, the H2 module was associated with a subset of clinical cartilage samples (“Clinical Group 2”, cor = 0.44, *p* = 1e−07). Across species, the consensus module hub genes were comparable (*CD53, ALOX5AP, NCKAP1L, IGFSB6, CYBB, LCP1, LAPTM5*); however, the connectivity pattern (those genes with the highest degree) of the modules differed between the species (Fig. 5a, b). The C5 consensus module demonstrated significant enrichment of protein–protein interactions indicating that the shared module had a functional significance (Fig. 5c). Overall, genes with membership of the H2 module had more sparse connections than the comparable module in the rat. There was moderate correlation of module membership values (*k*ME) for the genes in the R5 module with the human H2 module (cor = 0.44, *p* = 1.7e–12) sufficient to indicate that module hubs identified in the rat were also likely to be hubs in the equivalent human module. Top hub genes are given in Supplementary Data SD35. Together with the description of the C4 module, these findings suggested that expression of genes in the M2 meta-module was consistent with a degenerate or dysregulated chondrocyte phenotype in both rat and human whole cartilage.

### Module associated with normal rat cartilage absent from human data

Cartilage samples from a sham intervention in the rat (joint injections with isotonic saline) were strongly associated with the R12 module, where classical cartilage-associated hub genes were identified (*Col2a1*, *Acan*, *Comp*; Supplementary Fig. 7a). A similar module was not identified in the human samples; the rat R12 module had low preservation in the human network (summary *Z*-score = 5.9, −log10 *p* value = −7.6). In the human network, the cartilage hallmark *COL2A1* was in the unassigned module (H12, gray), indicating no strong association with a co-expression module. There was no evidence of differential expression of *COL2A1* across human age sample or clinical sample groups (Supplementary Fig. 7b).

### Modules classify clinical samples from independent human data sets

**Fig. 6:**
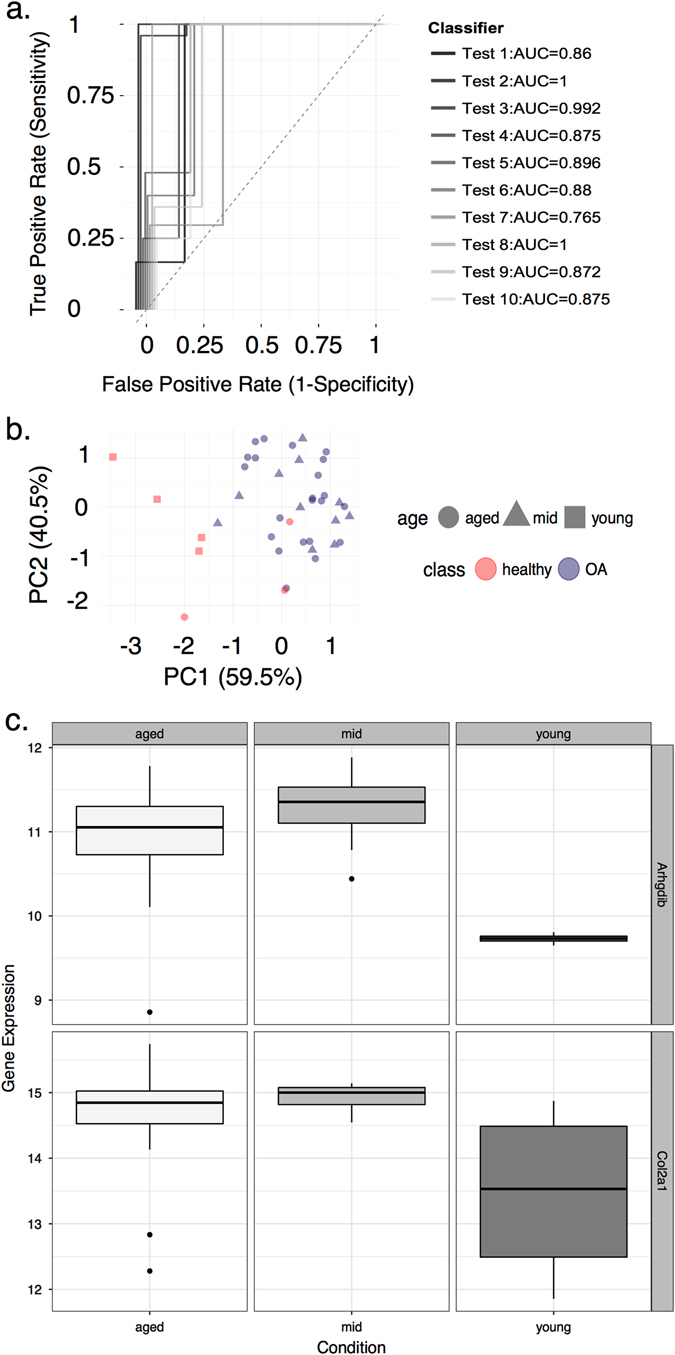
Development of an OA cartilage gene signature. **a** Receiver operator characteristic (ROC) curves for gene classifiers arising from 10 randomized test and training cohorts using data from the RAAK study, *n* = 40.^49^ *Plot* shows the true-positive rate (sensitivity, *y-*axis) against the false-positive rate (1-specificity, *x-*axis). Curves have been staggered at the origin (0,0) for clarity. The area under the receiver operator curve (AUROC), the capacity for a test to distinguish between two groups, is shown in the figure legend for each group of genes (Classifier). The ROC curve of the random classifier (AUROC = 0.5) is shown as a broken line bisecting the plot through the origin. In all cases, the AUROC for selected genes from the H4 module indicated that the probability that these classifiers would correctly rank healthy cartilage samples over OA samples was greater than the area under the curve of the random classifier. **b** Principal component analysis of gene expression data from two discriminatory genes (*ARHGDIB*, *RGS5)* derived from the human H4 module to exemplify the division of samples. Data arise from healthy (*n* = 7) and osteoarthritic (*n* = 33) cartilage samples (class) in young (16–18), mid-aged (>60), and aged (>70) individuals (see *key*). The first two principal components (PC1, PC2) described most of the variation between the young and mid-aged/aged samples. This combination of two genes had a representative low classification error (0.033) using 10-fold cross-validation as a robust estimate. Other combinations of genes were defined for each test, but in all cases *ARHGDIB* was the top-scoring gene (Supplementary Data SD42). **c** Expression of *ARHGDIB* in three age groups (*top panel*) was significantly different (*p* = 3.9e−05, Kruskall–Wallis test) with cartilage from young donors found to be lower. Expression of the cartilage hallmark gene collagen type II, *COL2A1*, (*lower panel*) was more variable across age groups (*p* = 3.7e−03)

Having established consensus modules associated with dysregulated chondrocyte phenotypes across species, a nearest shrunken centroid approach was used to define a minimal gene signature that predicted class membership for either healthy or osteoarthritic cartilage in humans. Genes from each human module were used as the selected features upon which to define a rule based upon gene expression. The H4 module (C4 consensus module, system development) discriminated well between healthy and OA samples as defined by values for the area under a receiver operator characteristic curve >0.5 (Fig. 6a) and as shown by principal component analysis (Fig. 6b). The G-protein signaling gene *ARHGDIB* (Rho GDP dissociation inhibitor beta) was consistently identified as the top-scoring gene in all iterations using H4 module genes (Supplementary Data SD42). Expression of *ARHGDIB* was found to be consistently lower in young and healthy cartilage samples from an independent data set (Fig. 6c), but it was not possible to distinguish between age and OA (cartilage health).

To define gene candidates with a temporal relationship with human cartilage degeneration, age-associated rat modules were considered. The R2 module was chosen as it was correlated with expression data from aged rats (Supplementary Fig. 5a), but had no association with in vivo intervention studies. The genes from the R2 module was used as the selected features as before. The most consistent high-ranking gene was *BCL6* (B-cell CLL/Lymphoma 6), a known senescence-associated gene (Supplementary Data SD42). This could discriminate between young and old human cartilage from an independent data, as before (Supplementary Fig. 8a–c). In young human cartilage *BCL6* was more highly expressed than older groups (*p* = 5.4e−3), but this relationship was also confounded by health status of cartilage with *BCL6* higher in healthy cartilage derived from young individuals. Expression of *Bcl6* across rat age groups demonstrated a reciprocal trend with cartilage from adult and aged rats showing higher expression relative to neonatal and young samples (*p* = 0.034). Other modules also showed evidence of low classification errors, but had not demonstrated strong associations with OA samples. With healthy cartilage arising from predominantly young individuals, the association between age and cartilage health confounded genes with classification potential.

## Discussion

This study demonstrates that added value may be gained from re-analysis of small transcriptomic studies using a network-based systems biology approach to establish conservation and divergence of transcriptional subnetworks between chondrocyte phenotypes in humans and a rodent model species. This study provides a comprehensive reference framework for chondrocytes in both physiological and pathological contexts. This has relevance to an understanding of the degenerative processes that underlies OA in humans and develops a clearer understanding of the utility of rodent models. Gene co-expression network analysis has become an increasingly useful tool to understand the regulatory context in which single genes may operate and has been applied to many aspects of human biology, but has not been used to determine the preservation of cross-species modules for OA.

A data-merging approach was employed in this study to agglomerate raw expression data from multiple sources. There is a strong rationale for the integration of microarray expression data including the increase in statistical power, re-use of rare specimens and animal data *in silico*, exploration of variance and noise in the data, and the definition of biological markers and prognostic signatures not evident in a small analysis.^18^ There is no definitive methodology for tackling microarray data merging, although integration at an interpretative level, using summary statistics, is a common approach. Data collection, curation, establishing common gene identifiers, and standardizing data-handling methods are essential.^19^ Integration is confounded by microarray platforms from different vendors being inherently incomparable.^20^ We chose to work with the probe-level, raw expression data from Affymetrix arrays using 25-mer probes to allow greater flexibility in our analysis; a *Z*-score global normalization strategy was used to avoid “over-smoothing” the expression data.

The validity of rodent models of OA at a systems level has not been established; this study sought to define shared mechanisms of cartilage degeneration between rats and humans. Two consensus modules (termed C4 and C5, comprising the M2 meta-module) were associated with perturbed cartilage phenotypes in human clinical samples and subsets of rodent OA models and sham surgical interventions. The C5 module was associated with immune system processes with a many highly connected module hub genes preserved between the species. Of these conserved hub genes *CD53, ALOX5AP*, and *NCKAP1L* have previously been identified, using a co-expression network analysis, as key drivers in other rodent inflammatory conditions,^21^ suggesting that degenerative processes in cartilage are likely to be associated with inflammatory regulatory networks already defined in other disease processes. Although a pro-inflammatory molecular mechanism associated with OA progression is clear,^22^ there is no definitive evidence that DNA polymorphisms in inflammatory genes are a risk factor for OA.^23^ This study reveals that inflammatory gene networks are conserved across species and that modules contain genes widely described as having an association with OA.

We show that a differentiation and systems development module is preserved across species and associated with subsets of cartilage samples. Specifically, the C4 consensus module was associated with skeletal system development, cellular differentiation, ECM annotations, and PI3K-Akt signaling. The presence of genes with known angiogenesis (*EMCN, KDR*), chondrogenesis, OA, and cartilage knockout phenotypes—including *DMP1*,^24^ *CTSK*,^25^ *MMP9*, and *ACP5* ^26^—in a single consensus module demonstrates the utility of a network-based systems biology approach to an understanding of a multigene disorder across species.

OA is a multifactorial and complex disease with diagnosis by imaging modalities usually in the late stages of joint degeneration. Critically, this degeneration occurs over a considerable duration over which intervention could occur; a lack of disease-modifying therapeutics and poor characterization of pre-osteoarthritic disease states means that early intervention is not possible.^27^ Clinical information from public repositories was limited with age, sex, and only a general description of cartilage health available. Additionally, no information on co-morbidities (e.g., obesity) is provided in public repositories for these samples. Notably, a range of expression profiles from osteoarthritic and ostensibly normal cartilage was apparent, and these samples did not group according to definitions of cartilage health made by gross appearance.

The M2 meta-module had the greatest overlap with the H4 module in the human network. A class prediction approach was used to define a gene signature, using member genes of the H4 module, to discriminate osteoarthritic cartilage from healthy samples. Classification analysis identified a G-protein signaling gene *ARHGDIB* (Rho GDP dissociation inhibitor beta, or *RhoGDI2*) as the highest scoring gene using an independent cohort; a variable signature of fewer than 25 genes could be used to consistently distinguish healthy from osteoarthritic cartilage. This indicated that a module of highly connected genes established as being shared across species was also discriminatory between healthy and OA cartilage samples in an independent data set. Notably, expression of ARHGDIB protein has been found to be differentially abundant between healthy and late-stage OA synovial fluid.^28^ Subgroups of clinical samples showing differential associations with modules may represent different stages in cartilage degeneration, including pre-OA states,^27^ and should be investigated further as potential diagnostic and prognostic markers.

Although a strong risk factor, ageing alone does not cause OA, but promotes OA in conjunction with a number of other risk factors, e.g., obesity, through multifaceted mechanisms that are incompletely understood.^29, 30^ Age is a confounding variable in this analysis, with the majority of human studies profiling OA cartilage from aged patients. In the class prediction analysis from independent data ostensibly normal cartilage from aged donors is shown to cluster more readily with OA samples. We attempted to tease apart this association by using genes associated with aged rat cartilage for further class prediction analysis. The senescence and age-associated gene *BCL6* ^31^ was identified, but high expression was found in both young and healthy cartilage. Ageing chondrocytes do exhibit an associated decline in synthetic capacity (so-called “chondrocyte senescence”) but retain the ability to produce pro-inflammatory cytokines.^32, 33^ Functionally, this correlates with the M2 meta-module where consensus hubs are associated with immunomodulation or cell differentiation. Therefore, in human samples it is not possible to distinguish module associations with the pathophysiological process of OA from ageing per se in part because of the bias in healthy tissue arising only from young individuals.

A module containing the hallmarks of functional cartilage (R12, *Col2a1, Acan, Comp, Hapln)* was found in the rat co-expression network associated with sham controls (isotonic saline injection into normal joints). These genes were not assigned to a distinct module in the human, and *COL2A1* was not differentially expressed across samples. If the assumption is made that the R12 module represents a healthy, functional cartilage profile, the absence of an equivalent module in human data may indicate that reported cartilage control tissue in most human transcriptomic surveys is not functionally normal or expression of *COL2A1* is not the best indicator of human cartilage degeneration. The lack of age-matched healthy and OA human cartilage gene expression profiles is a notable obstacle to further interpretation of the results.

Given the complexity of this type of analysis, the key points of this study may be reduced to the following: (i) the use of *ARHGDIB* as a potential OA marker or therapeutic target should be explored further, given the existing OA association, with *BCL6* expression validated as a marker of chondrocyte senescence or age-associated cartilage degeneration; (ii) the accessibility of normal human cartilage needs to be evaluated as there is a dearth of healthy samples and clinical data in public repositories; (iii) sham surgery and surgical destabilization of the joint in rat models of OA may be poor comparators, given the co-clustering of samples in this study; (iv) community-based approaches in OA research are essential to developing appropriate standardized in vivo models in particular full experimental disclosure is lacking in many of the rat studies; (v) hub genes are the fragile points in a network; a number of these are indicated for conserved modules with OA associations. These should be considered as novel knockout targets in the mouse as part of age-matched longitudinal studies; (vi) further validation of co-expression networks with phenotypic and quantitative traits should be undertaken to elucidate causal mechanisms.

To conclude, two highly correlated consensus modules are conserved across species when cartilage gene expression profiles are considered. Inflammation and differentiation status of the resident chondrocytes are shown to be strongly associated with a dysregulated cartilage phenotype in both humans and rats. While evidence for an association with a number of established OA genes is present, demonstration that these OA-associated genes are co-expressed has not previously been shown. We found that some elements of human OA are conserved in rodent models, but suitably matched prospective studies of sufficient power across species are required to maximize translational impact and utility in the discovery of disease-modifying therapeutics to target multiple disease-associated networks.

## Methods

### Data collection, merging, and standardization

An overview of the general approach used for data collection and analysis is provided in Supplementary Figs. 1 and 2. Gene expression profiles were selected from curated public-access repositories Gene Expression Omnibus (http://www.ncbi.nlm.nih.gov/geo/)^34^ and ArrayExpress (http://www.ebi.ac.uk/arrayexpress/).^35^ To be included in initial analysis studies had to: (a) be performed in the rat (*Rattus norvegicus*) or human, (b) profile chondrocytes from tissue or in vitro culture systems, (c) provide adequate phenotypic information, (d) provide complete raw data for a minimum of three biological replicates, (e) be performed on Affymetrix microarray platforms (Affymetrix^®^ Inc., USA) using 25-mer oligo probe sets. All studies released up to December 2015 were considered. All raw data were imported into and analyzed using *R*.^36^ A quality control and pre-processing pipeline was applied to each autonomous study, and these assessed for systematic technical issues. Expression data were background-corrected using the RMA algorithm^37^ with cyclic loess normalization method applied across each data set. Probe sets were re-annotated with the appropriate Ensembl gene identifier. Expression data for each gene were aggregated and collapsed into a single-gene measurement consisting of the maximum mean expression value using the “collapseRows” function in the WGCNA.^38^ The output of this workflow was a normalized matrix of expression values consisting of one summarized gene per row. Intersection of data sets by common gene identifiers was performed such that all data sets contained the same gene identifiers. The matrix of merged data sets was termed a “meta-set”. The rat meta-set consisted of 115 arrays (10,159 common annotations) and the human meta-set consisted of 129 arrays (11,392 common annotations). A *Z*-score normalization was applied to each species meta-set using the inSilicoMerging *R* package.^39^ A complete description of data sources and retained samples is provided in Supplementary Data SD43 (human) and SD44 (rat).

### Weighted gene co-expression network analysis

To establish universal gene identifiers and facilitate comparison across species, rat gene identifiers from Affymetrix probes were re-annotated with human Ensembl gene orthologs using biomaRt, an *R* interface with the Biomart database (www.biomart.org).^40^ Only identifiers that were common to both meta-sets were retained and genes with a global variance less than 0.3 were removed to reduce noise and computational demands. The data met the assumptions of a scale-free network. The general approach employed to develop co-expression modules is described by Miller et al.^16^ and is outlined in graphical workflows in Supplementary Figs. 1 and 2. A consensus network represents a single network arising from multiple sources of data constructed from the weighted average of correlation matrices from both the human and rat in this study. By definition, consensus modules are the branches of a clustering tree developed from a consensus gene dissimilarity, comparable to the single-network approach; consensus modules contain genes that are closely related in both networks, i.e., the modules are present in both networks.^41^ Consensus network and module generation were performed in WGCNA (version 1.49) with the following changes to the default settings for consensus network generation: *β* = 7, deepSplit = 1, cutHeight = 0.25, and a minimum module size of 30 genes. This was consistent with the parameters used for single-species network generation. Only consensus module colors are equivalent across the species. Modules were characterized based upon a representative gene termed the “ME defined as the first principal component of the module gene expression profile. MEs were used for module–trait association analysis, differential eigengene network analysis, and for differential gene expression analysis. Difference in expression across trait groups was tested using a Kruskall–Wallis one-way analysis of variance. A gene's module membership (*k*_ME_) is defined as the Pearson correlation between each gene and each ME; genes with high *k*_ME_ values were considered “hub” genes and were highly co-expressed within a subnetwork. How well these hubs were preserved across species determined by correlating gene *k*_ME_ values between species.

Module preservation statistical tests^42^ were used to assess how well network properties of a module in one reference data set were preserved in a comparator data set (modulePreservation function in WGCNA). Preservation statistics are influenced by a number of variables (module size, network size, etc). A composite preservation *Z*-score (*Z*_summary_) was used to define preservation relative to a module of randomly assigned genes where values 5 > *Z* < 10 represent moderate preservation, while *Z* > 10 indicated high preservation. The composite statistic summarized density-based and connectivity-based preservation statistics (Eq. 1):

1 $${Z_{{\rm{summary}}}} = \frac{{{Z_{{\rm{density}}}} + {Z_{{\rm{connectivity}}}}}}{2}$$

Density-based measures assessed whether module nodes remained densely connected in a test network; connectivity-based measures defined whether intranode connectivity patterns in the reference network were similar to those in the test network. A separate summary *p* value for module preservation, given as the median of the log-*p* values for the associated permutation *Z* statistics, was calculated. Permutation tests, where the module labels of the test network were randomly permuted, were employed to determine the significance of the observed preservations test statistics. A module of randomly assigned genes, “gold” (R21) module, was prepared as a sham module to evaluate bias in the module preservation across species. The reader is referred to other sources for glossaries of terms associated with WGCNA.^16, 42^

The gene expression profile for a consensus module of highly co-expressed genes could be summarized by a single representative gene, the eigengene (described as the first right-singular vector of the standardized expression profile for each module), i.e., a module could be characterized by a single representative gene.^41^ Eigengene networks for single (species-specific) co-expression networks were prepared using the correlations exhibited by pairs of eigengenes from different modules where the connection strength (adjacency) between eigengenes (*E*) *I* and *J* (Eq. 2):

2 $${a_{{\rm{Eigen,}}IJ}} = \frac{{1 + {\rm{cor}}\left( {{E_I},{E_J}} \right)}}{2}$$

The study considered the correlation preservation between all pairs of consensus MEs across the two species networks, A_Eigen_^(human)^ and A_Eigen_^(rat)^, where A_Eigen_^*(s)*^ is the adjacency matrices for data set (*s*) defined in Eq. 2. A *preservation network* Preserv^(human,rat)^ = Preserv(A_Eigen_^(human),^ A_Eigen_^(rat)^) was prepared in which adjacencies are defined as:

3 $${\rm{Preserv}}_{IJ}^{{\rm{human,rat}}} = 1 - \frac{{\left| {{\rm{cor}}\left( {E_I^{{\rm{human}}},E_J^{{\rm{human}}}} \right) - {\rm{cor}}\left( {E_I^{{\rm{rat}}},E_J^{{\rm{rat}}}} \right)} \right|}}{2}$$

where $E_{\rm{I}}^{\left( {\rm{s}} \right)}$is the eigengene of the I-th module in the data set *s*. High values of ${\rm{Preserv}}_{{\rm{IJ}}}^{{\rm{human,rat}}}$ indicated robust preservation, across the two networks, of the correlation between module eigenegenes I and J. The scaled connectivity *C*_I_, or degree, for the I-th module (Eq. 4) is described as the mean connection strength with all other eigengenes; the scaled connectivity of the preservation network is given by:

4 $${C_I}{\rm{Preserv}}_{IJ}^{{\rm{human,rat}}} = 1 - \frac{{\mathop {\sum}\nolimits_{J \ne I} {\left| {{\rm{cor}}\left( {E_I^{{\rm{human}}},E_J^{{\rm{human}}}} \right) - {\rm{cor}}\left( {E_I^{{\rm{rat}}},E_J^{{\rm{rat}}}} \right)} \right|} }}{{2\left( {N - 1} \right)}}$$

(where *N* denotes the number of MEs); this value is found to be close to 1 if there is preservation of the correlation between the I-th eigengene and all other eigengenes across the two networks. The density of the eigengene network *D*(Preserv^(human,rat)^) (Eq. 5), defined as the average scaled connectivity, is given by:

5 $$D\left( {{\rm{Preser}}{{\rm{v}}^{\left( {{\rm{human,rat}}} \right)}}} \right) = 1 - \frac{{\mathop {\sum}\nolimits_I {\mathop {\sum}\nolimits_{J \ne I} {\left| {cor\left( {E_I^{{\rm{human}}},E_J^{{\rm{human}}}} \right) - {\rm{cor}}\left( {E_I^{{\rm{rat}}},E_J^{{\rm{rat}}}} \right)} \right|} } }}{{2N\left( {N - 1} \right)}}$$

Values of *D*(Preserv^(human,rat)^) that are large, approaching 1, indicate strong preservation of correlation between all the eigengene pairs across the two networks (human and rat). Procedures to detect modules in networks could be applied to eigengene networks to find modules of highly positively correlated eigengenes, term “meta-modules”.^41^

### Module–trait relationships

To determine whether modules were associated with chondrocyte phenotypes or traits the MEs were correlated with a binary matrix coding, the membership of an individual sample to a phenotypic trait or experimental group (1 = member, 0 = non-member). Multidimensional scaling plots of each meta-set was used to define clusters of samples rather than using the phenotypic data from the published data set to define sample groups.

### Network visualization and annotation

The module network structure, consisting of nodes (genes filtered for high module membership, *k*ME) and edges (weighted intramodular connections based upon the topological overlap matrix) were represented graphically using Cytoscape (v3.3.0, January 2016).^43^ Only nodes with high degree were retained for clarity. Enrichment of protein–protein interaction networks was assessed using STRING v10 (http://string-db.org/).^44, 45^ Pathway enrichment analysis was undertaken for each consensus module using the ConsensusPathwayDB platform (release 31 September 2015) (http://cpdb.molgen.mpg.de).^46^ Modules were functionally annotated using DAVID (https://david.ncifcrf.gov).^47^

### Class prediction analysis

Class prediction analysis was performed using the pamr package implemented in *R* ^48^ (see Supplementary Fig. 3). This method employs a “nearest shrunken centroids” approach to determine cohorts of genes that best characterize classes from high dimensional data. The average gene expression for every gene in a class is divided by the within-class SD for the gene; this is the standardized centroid for each class. The gene expression profile of a new (test) sample is compared to the class centroid in nearest centroid classification; the predicted class for the new sample is the nearest class centroid by squared distance. The nearest shrunken centroid modification “shrinks”, by a threshold value, all class centroids toward an overall centroid; the threshold is defined by a 10-fold cross-validation for a range of threshold values. Genes from modules with important trait associations were used as the selected features for class prediction where the two classes were “healthy” or “osteoarthritic” cartilage. Classification training was performed on gene expression data (Illumina) from an independent data set^49^ profiling healthy (*n* = 7) and osteoarthritic (*n* = 33) cartilage. This was repeated for each of ten randomized test and training sets. Receiver operator characteristic (ROC) curves and area under the curveanalysis was undertaken using the ROCR package in *R* for each gene signature.^50^

### Code availability

Code contributing to the analysis presented here is available in Supplementary Methods 1 with supporting processed and annotated data files for rat and human.

## Electronic supplementary material

Supplementary Material

Supplementary Material
