# Supplementary material for: Cross-species gene modules emerge from a systems biology approach to osteoarthritis
Source: NPJ Syst Biol Appl. 2017 May 17;3:13. doi: 10.1038/s41540-017-0014-3 (PMC5460168; doi:10.1038/s41540-017-0014-3)
Supplement: Supplementary file 1 — Supplementary Material [file 41540_2017_14_MOESM1_ESM.docx]

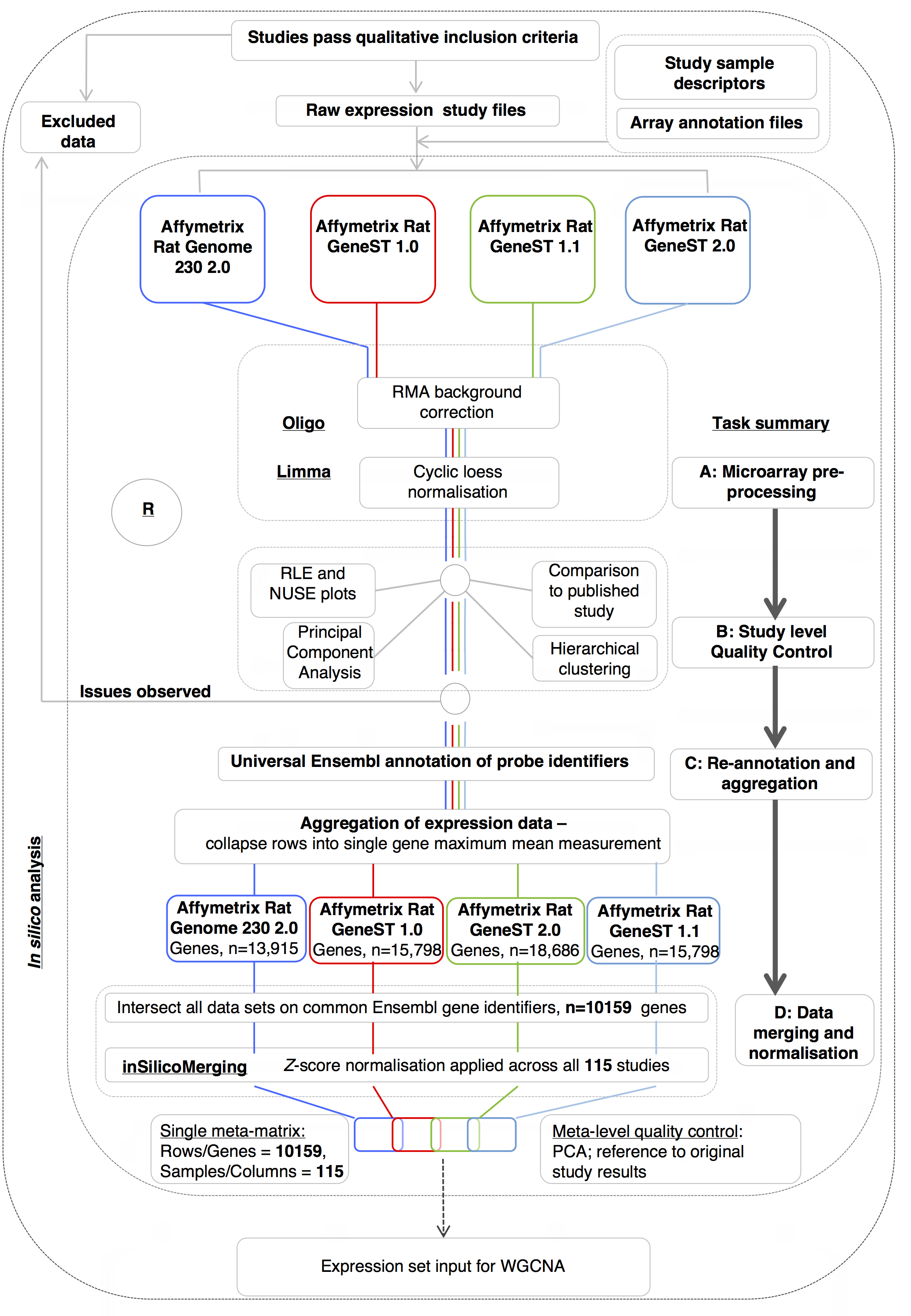


**Supplementary Figure 1**

**Supplementary Figure 2**


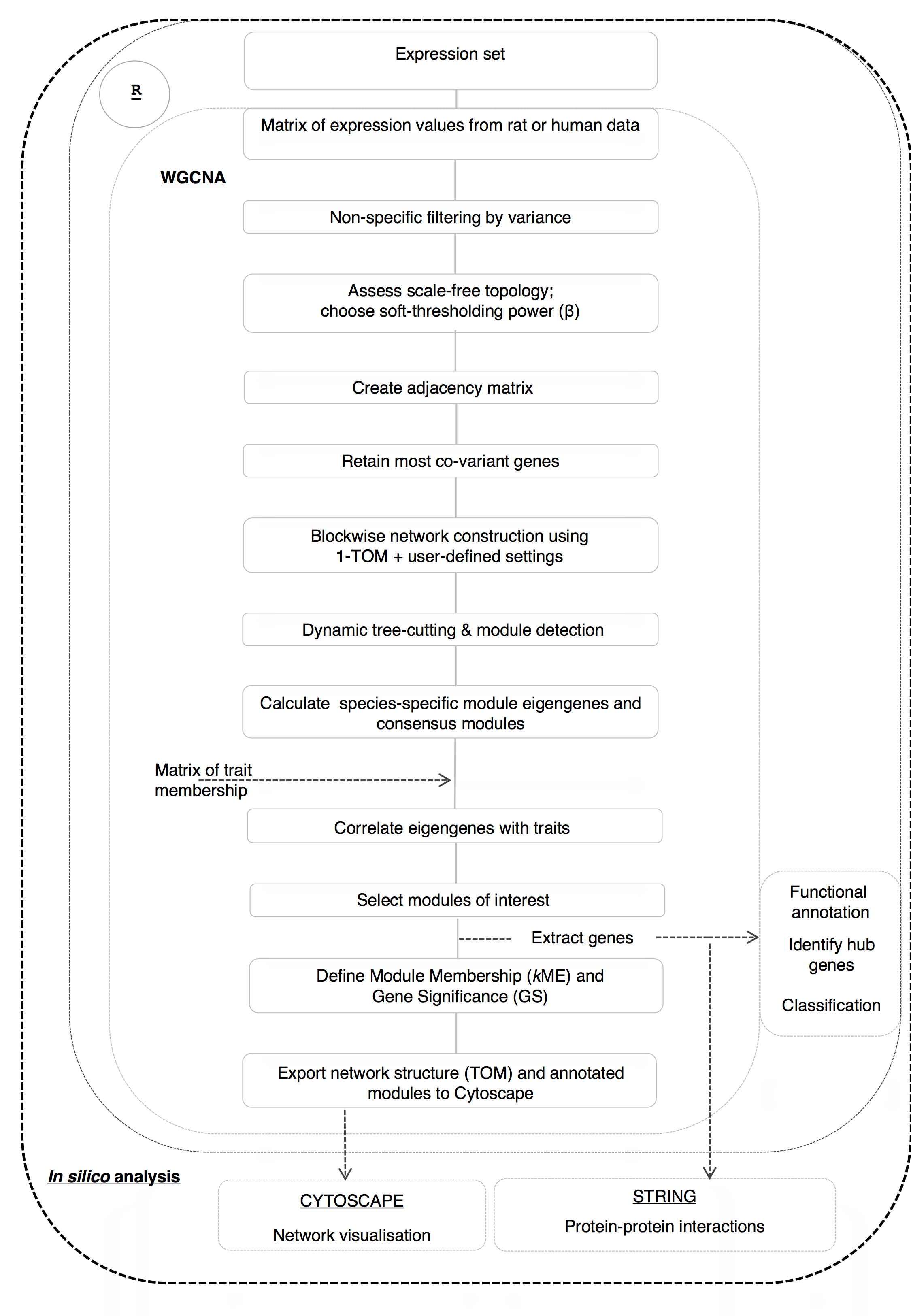


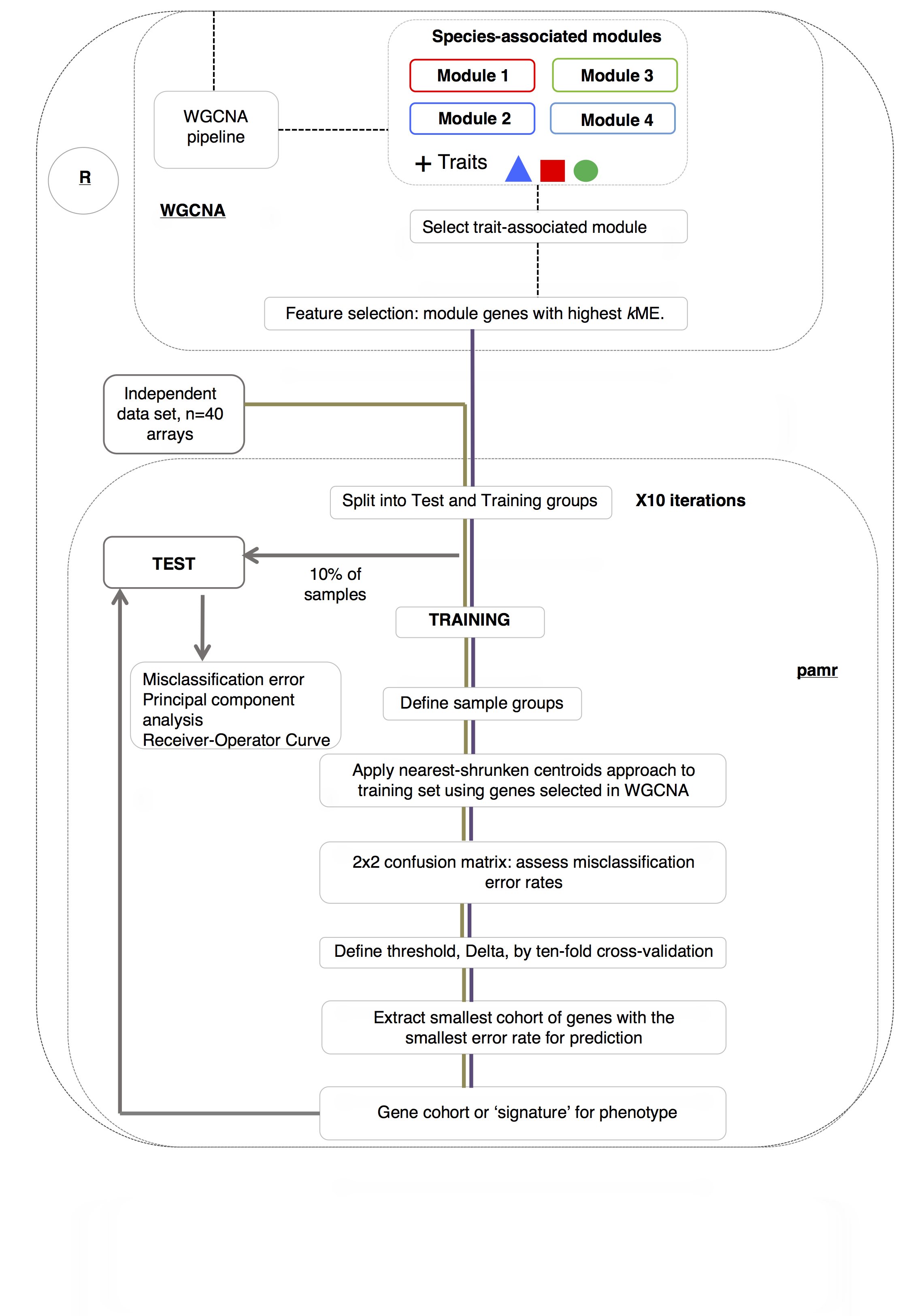


**Supplementary Figure 3**


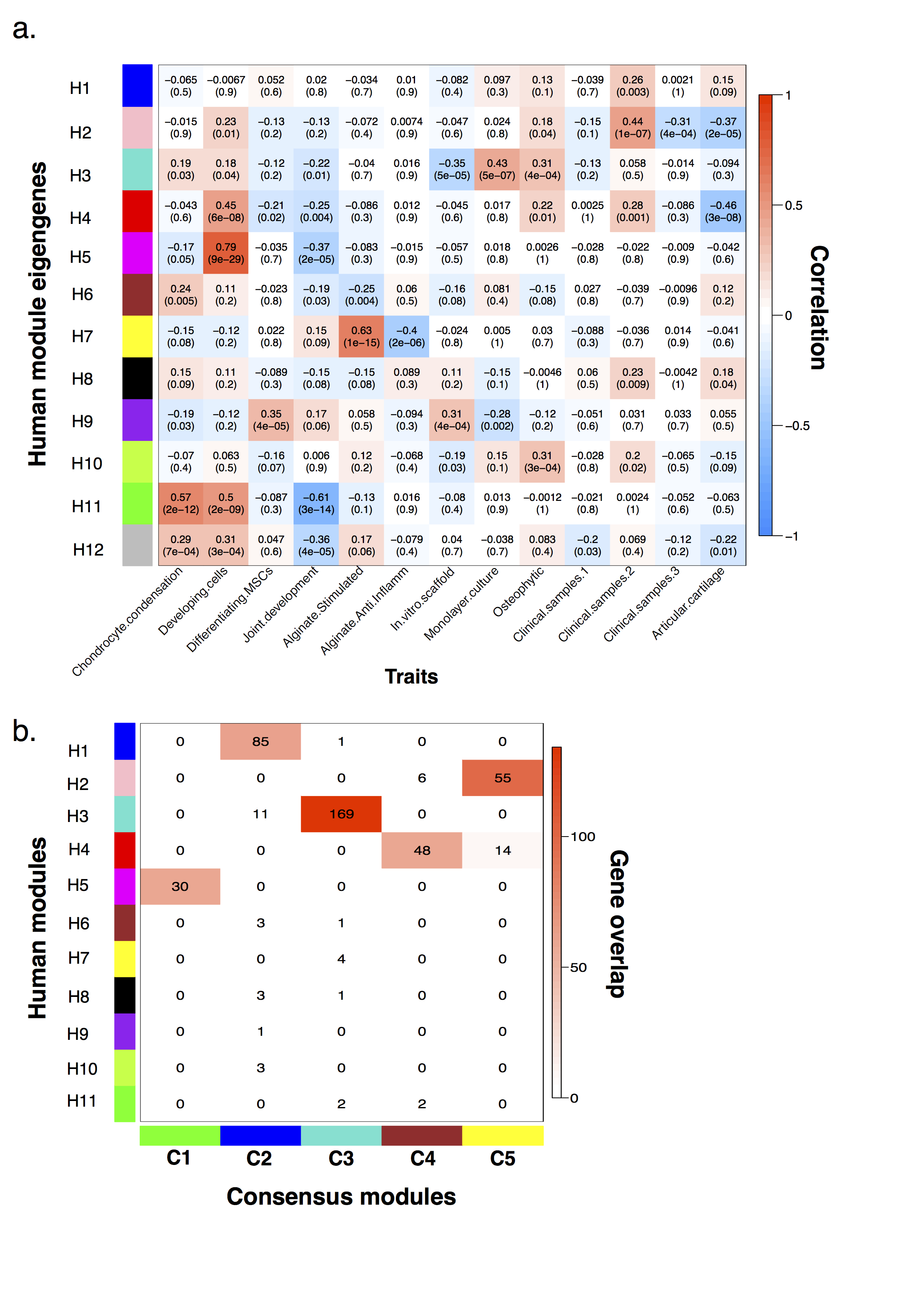
**Supplementary Figure 4**


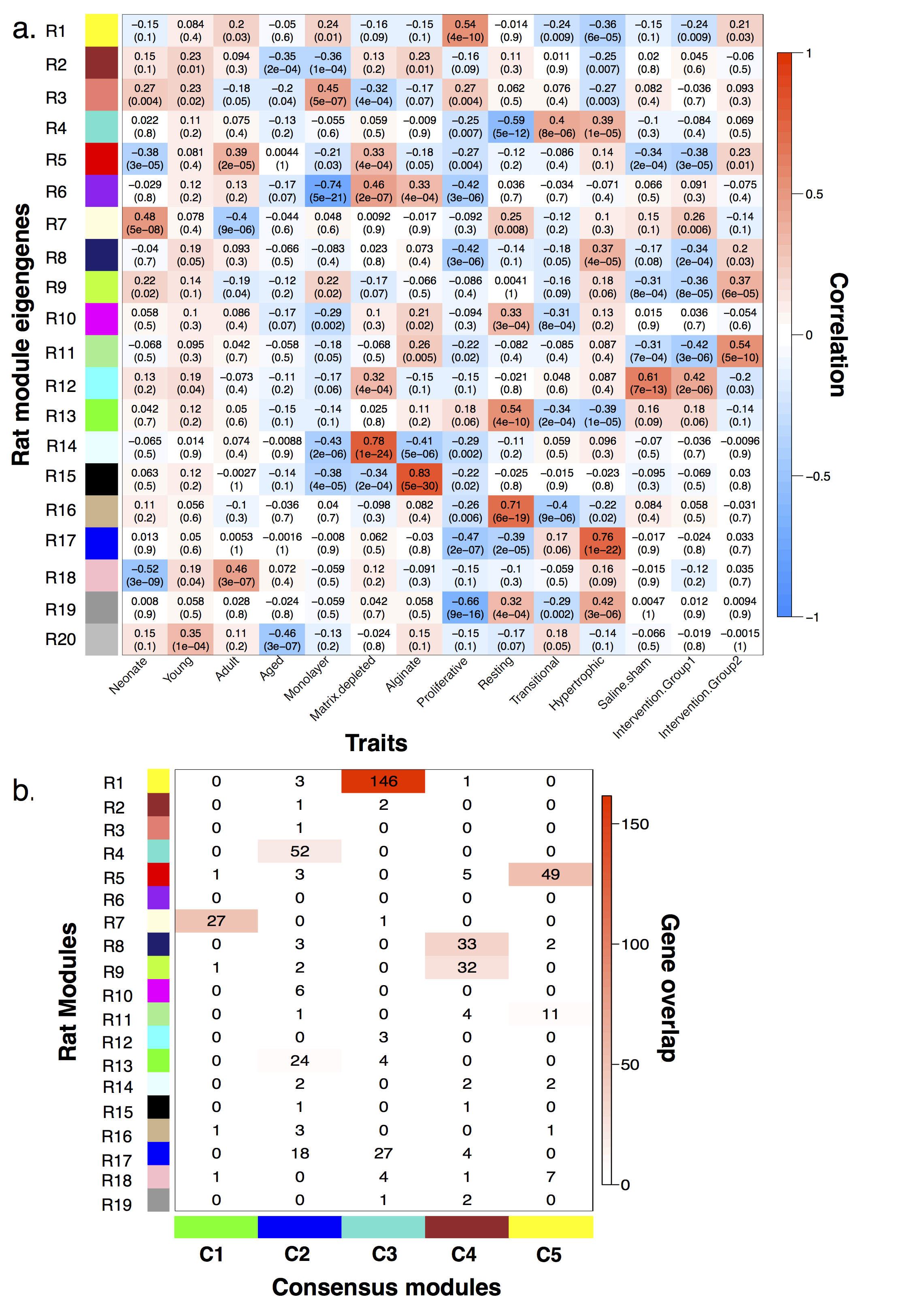


**Supplementary Figure 5**


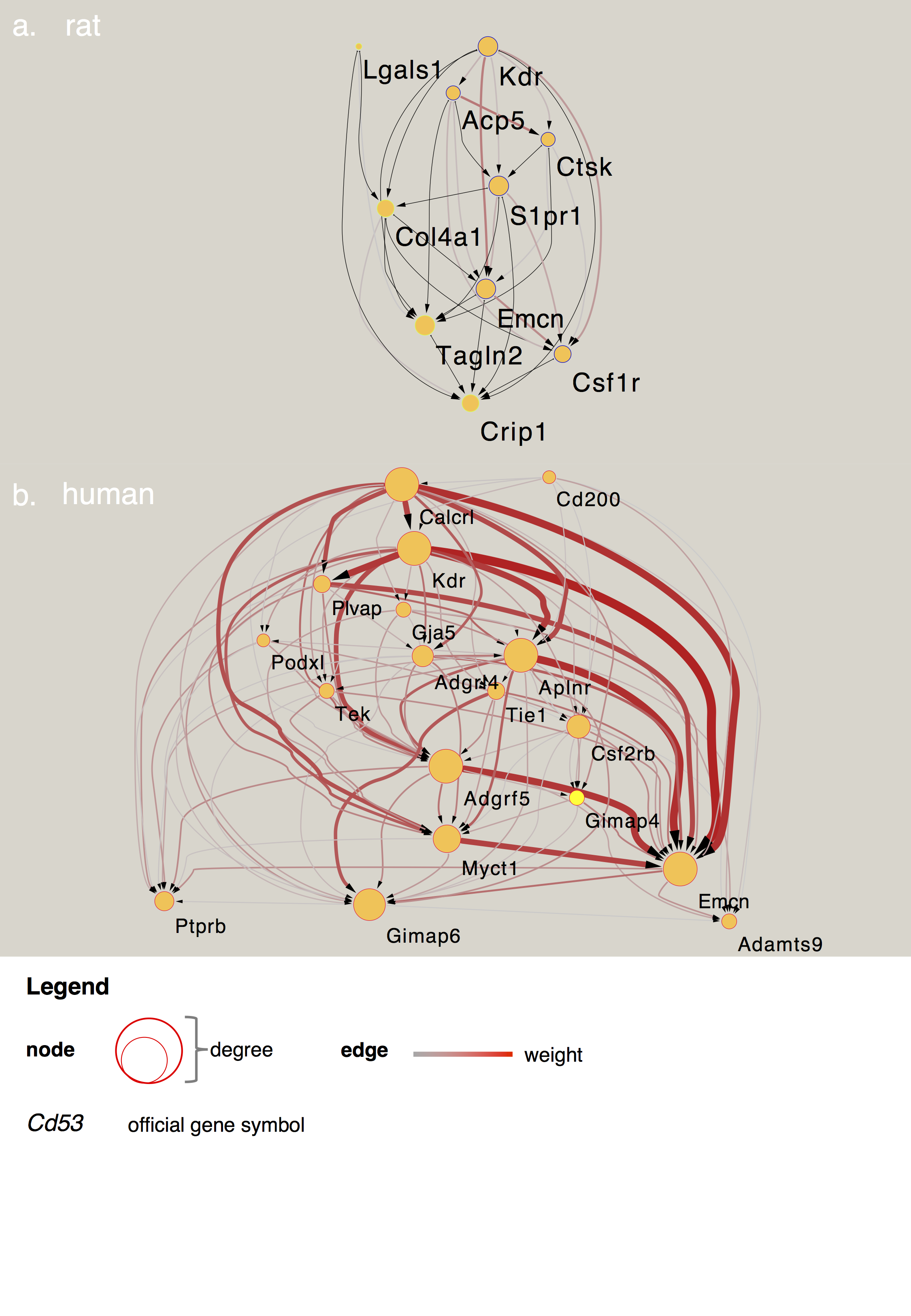


**Supplementary Figure 6**


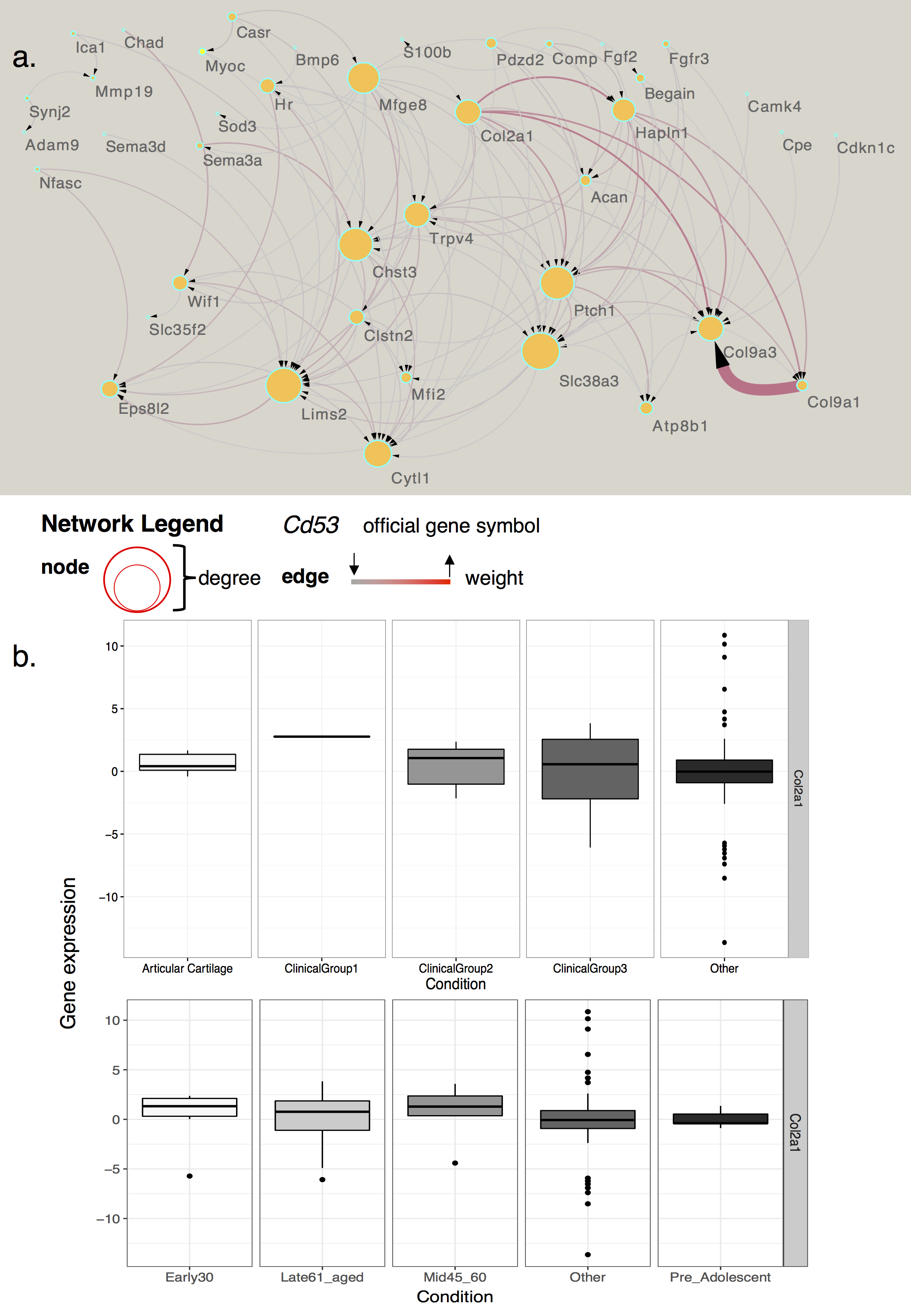


**Supplementary Figure 7**


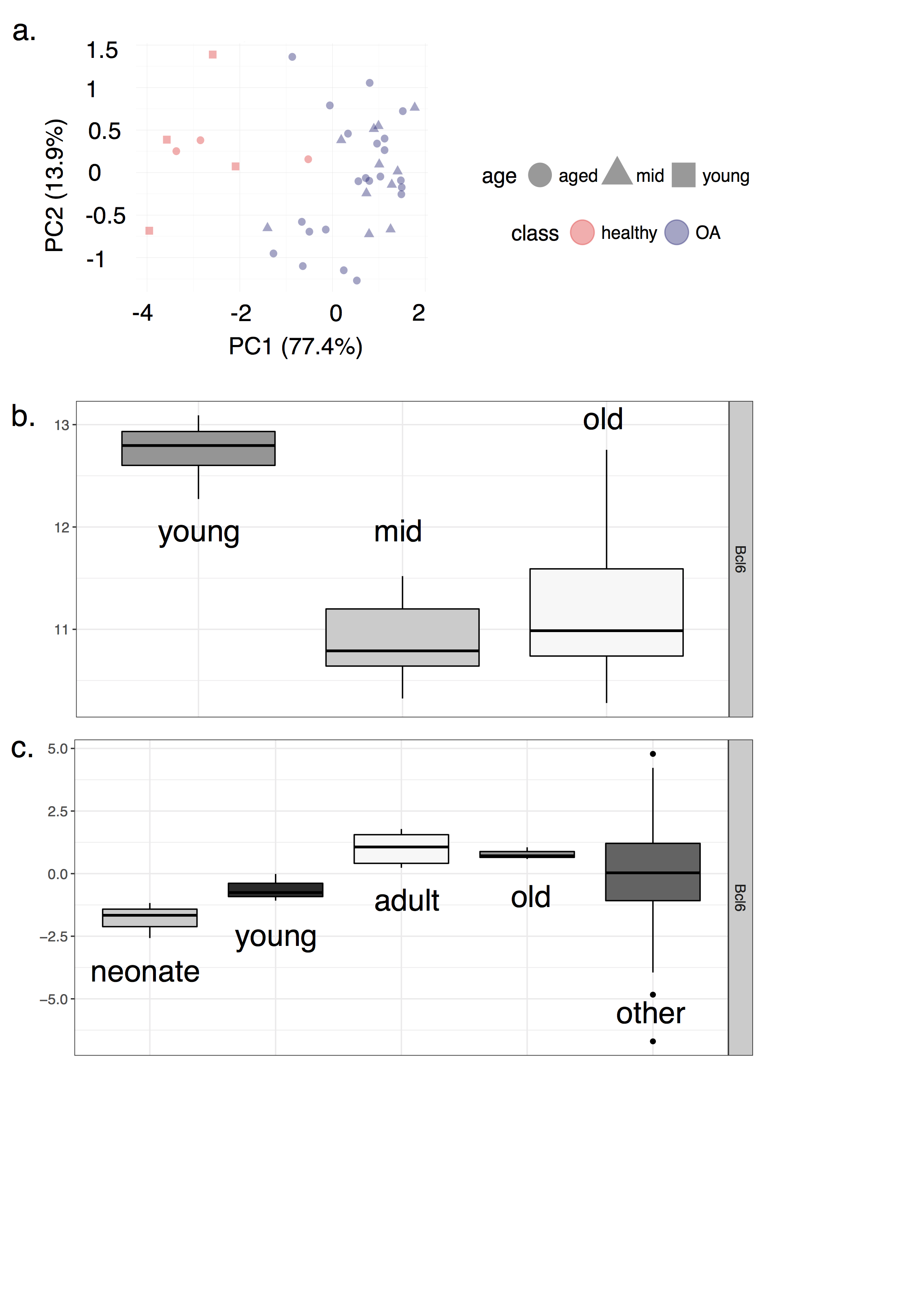


**Supplementary Figure 8**

| Rat  Module \|  Colour \| Genes in module | Module Preservation  (z-score, Bonferroni -log10 p-value) | Consensus Gene Ontology  BP \| MF \| CC \| KEGG pathway  (Bonferroni p-value <0.05) |
| --- | --- | --- |
| R1 \| Yellow (311) | 35.2 (-326.7) | Cell cycle, DNA metabolic process, microtubule cytoskeletal organisation \| nucleotide binding \| Intracellular part, nuclear part, chromosome \| DNA replication |
| R2 \| Brown (343) | 15.2 (-50.9) | RNA metabolic process; protein modification by small protein conjugation or removal \| RNA binding \| intracellular part; nuclear lumen \| Ubiquitin mediated proteolysis |
| R3 \| Salmon (156) | 13.9 (-47.6) | No significant BP annotation \| transferase activity, transferring glycosyl groups \| endoplasmic reticulum; endomembrane system; cytoplasmic part \| no significant KEGG annotation |
| R4 \| Turquoise (452) | 12.1 (-37.9) | Sterol biosynthetic process; positive regulation of protein ubiquitination; primary metabolic process \| pyrophosphatase activity \| cytoplasm; mitochondrial matrix \| Sterol biosynthesis; proteosome |
| R5 \| Red (234) | 10.9 (-27.4) | Immune system process, defense process, cell activation \| signal transducer activity, protein kinase activity, protein homodimerization activity\| plasma membrane part, cell surface\| Cell Adhesion Molecules |
| R6 \| Purple (175) | 10.2 (-24.6) | No significant BP annotation \| No significant MF annotation \| Intracellular non-membrane-bounded organelle; intracellular \| No significant KEGG annotation |
| R7 \| Light.yellow (69) | 10.1 (-30.4) | Muscle system process; regulation of muscle contraction; muscle organ development \| structural constituent of muscle; cytoskeletal protein binding \| contratile fibre; striated muscle thin filament \| Cardiac muscle contraction |
| R20 \| Grey \| (2075) | 9.69 (-29.2) | Unassigned genes. |
| R8 \| Midnight.blue (108) | 8.97 (-22.3) | System development, biomineral formation, skeletal system development \| No significant MF annotation\| Extracellular region part, plasma membrane, extracellular matrix \| No significant KEGG annotation |
| R9 \| Green.yellow (161) | 8.86 (-18.9) | Cell adhesion; anatomical structure development; cell differentiation; regulation of cell motion \| calmodulin binding; extracellular matrix structural constituent \| extracellular region; extracellular matrix; contractile fibre \| Focal adhesion |
| R10 \| Magenta (202) | 7.8 (-14.9) | RNA metabolic process; chromatin modification \| No significant MF annotation \| nuclear part; intracellular \| No significant KEGG annotation |
| R11 \| Light.green (86) | 6.6 (-9.6) | Response to wounding, regulation of immune system process \| endopeptidase activity \| extracellular region, lytic vacuole \| No significant KEGG annottaion |
| R12 \| Cyan (114) | 5.89 (-5.12) | Multicellular organismal development; cell differentiation; skeletal system development; cartilage development \| glycosaminoglycan binding \| extracellular region; extracellular matrix \| No significant KEGG annottaion |
| R13 \| Green (244) | 4.9 (-5.12) | No significant BP or MF annotation \| intracellular part; intracellualar organelle lumen \| No significant KEGG annotation |
| R14 \| Light.cyan (94) | 3.91 (-5.24) | No significant BP annotation \| Receptor binding \| Extracellular space \| No significant KEGG annotation |
| R15 \| Black (208) | 3.6 (-3.5) | Response to chemical stimulus, system development, organ regeneration, regulation of developmental process \|no significant annotation for MF, CC, or KEGG |
| R16 \| Tan (161) | 3.1 (-1.95) | Skeletal system development, ossification, embryonic morphogenesis, cell adhesion \| No significant MF, CC, or KEGG annotations |
| R17 \| Blue (449) | 2.4 (-3.5) | No significant annotations BP \| transferase activity, kinase activity \| Cytoplasm \| No significant KEGG annotations |
| R18 \| Pink (202) | 2.2 (-0.57) | Immune system process \| No significant MP annotation \| plasma membrane part \| Porphyrin and chlorophyll metabolism |
| R19 \| Grey60 (89) | 0.91 (0) | No significant annotations |
| R21 \| Gold (100) | 1.88 (-0.4) | Sham module of randomly selected genes. No significant annotations |

**Supplementary Table 1**

| Human Module \| Colour \| Genes in module | Consensus Gene Ontology  BP \| MF \| CC \| KEGG pathway  (Bonferroni p-value <0.05) |
| --- | --- |
| H1 \| Blue (369) | Generation of precursor metabolites and energy; oxidative phosphorylation \| oxidoreductase activity \| cytoplasm \| Oxidative phosphorylation |
| H2 \| Pink (77) | Positive regulation of immune response; cell activation \| molecular transducer activity \| plasma membrane \| No significant pathways |
| H3 \| Turquoise (744) | Cell cycle; DNA replication \| nucleotide binding \| intracellular; nuclear part; spindle \| DNA replication |
| H4 \| Red (122) | Organ development; cell differentiation; signal transduction \| signal transducer activity \| plasma membrane part; No significant pathways |
| H5 \| Magenta (73) | Organ development; muscle system process \| regulation of muscle contraction \| contractile fibre; actin cytoskeleton \| No significant pathways |
| H6 \| Brown (213) | No significant annotations |
| H7 \| Yellow (166) | Response to external stimulus; response to wounding; inflammatory response \| cytokine activity \| cell fraction; extracellular space \| Cytokine-cytokine receptor interaction |
| H8 \| Black (111) | Translation; co-factor metabolic process \| no significant MF terms \| cytoplasmic part; mitochondrion \| No significant pathways |
| H9 \| Purple (47) | Cellular amino acid process \| Ligase activity \| soluble fraction; cell fraction \| Aminoacyl-tRNA biosynthesis |
| H10 \| Green.yellow (33) | Response to virus; proteolysis \| No significant MF annotations \| Cytoplasm \| RIG-I-like receptor signalling pathway |
| H11 \| Green (127) | Multicellular organismal development; cell projection organisation \| No significant MF, CC, or KEGG annotations |
| H12 \| Grey (3794) | Unassigned genes |

**Supplementary Table 2**

**SUPPLEMENTARY FIGURE LEGENDS**

**Supplementary Figure 1: Data collection and pre-processing workflow** - Input data is derived from publically available gene expression studies (microarrays) performed in either the human or rat. Figure represents data collection and processing for rat Affymetrix microarray gene expression data (four different vendor platforms), but the same workflow was used for human data. Each independent study (see **Supplementary Data SD43-44** for all study descriptions) underwent the same workflow up to the point of data merging. Raw expression data and sample annotations were collected and underwent qualitative assessment for inclusion. A series of pre-processing steps were undertaken with the R programming environment, to establish the integrity of the data using well-defined protocols (R packages - limma, oligo). Affymetrix probes were reannotated with Ensembl gene symbols; for the rat these were converted to human gene orthologs. Expression data for each individual data set was aggregated into a single measurement for each gene (collapseRows function, WGCNA). Expression data from each Affymetrix platform was intersected on common Ensembl gene identifiers. Data sets were merged and a *z*-score global normalisation was applied to all studies to generate a single meta-matrix (rows = genes, columns= samples) to serve as the input for gene co-expression network analysis.

**Supplementary Figure 2: Data analysis pipeline using a suite of functions within the WGCNA package.** Pipeline represents the analysis undertaken for each gene expression data set (rat or human) and includes consensus module detection. An *n* × *n* similarity matrix, *S=*[*s_ij_*], describes the pairwise co-expression relationships between the genes *i* and *j* (Eq. 6), such that:

s*_ij_* = |*cor(i,j)*| Eq. 6.

Using an adjacency function the similarity matrix is converted to a symmetrical adjacency matrix, *A*=[a*_ij_*] which encodes the connection strength between pairs of nodes, such that a*_ij_* ∈ [0,1]. Diagonal elements of *A* equal 1, i.e a*_ii._* This matrix is used to define node connectivity. The genes with the strongest connections were retained to reduced computational demands. Measures of node dissimilarity are defined from a topological overlap matrix (TOM), 1-TOM. Average linkage hierarchical clustering used on the dissimilarity measures to detect modules. A dynamic tree-cutting algorithm is used to define gene modules. Module networks may be reduced to module representatives, module eigengenes, the correlations between which may be used to form an eigengene network. Networks are constructed for each data set in turn and species-specific functional annotations and module-trait associations are investigated. Consensus modules detection was employed to find modules that are common to both data sets. A consensus dissimilarity measure and average linkage hierarchical clustering was used to detect consensus modules. The consensus module eigengenes represented their corresponding modules and the correlations between these were used to construct a consensus eigengene network. Differential eigengene network analysis was performed to defined whether relationships between eigengenes were retained between the two data sets. Genes with high module membership and highly significant for traits were exported for functional annotation, network visualization using Cytoscape, and protein-protein interaction analysis using STRING.

**Supplementary Figure 3: Discriminating between healthy and osteoarthritic cartilage.** Data manipulation and analysis pipelines for class-prediction using nearest shrunken centroids method implemented in the pamr R package. Genes were filtered for ‘feature-selection’ by restricting available genes to those found within a module with strong trait associations. Gene signatures identified were used to classify test data. Misclassification error rates and areas under receiver-operator curves defined the predictive success of each signature. Expression data from an independent data set ^49^ was used as the source of test and training samples against which to create a discriminatory gene signature for healthy and osteoarthritic cartilage.

**Supplementary Figure 4:** **Human module-trait associations -** **a**, Module eigengenes were correlated with cartilage and chondrocyte phenotypes and clinical traits including clinical samples and *in vitro* studies. Matrix cells show absolute correlation values (top line) and associated *p*-values (bottom line). Colour intensity (right side vertical bar) varies with positive or negative correlation between a trait and module eigengene. For example, chondrocytes in alginate beads with cytokines (‘Alginate stimulated’) were strongly correlated with the H7 (yellow) module eigengene, whereas alginate beads treated with anti-inflammatory drugs (‘Alginate.Anti.Inflamm’) had a negative correlation with the same module, which is annotated for ‘cytokine activity’ and ‘inflammatory response’. Clinical samples did not cluster in groups based upon published patient data (sex, age, OA score, gross pathology) and were assigned to groups (‘Clinical Groups 1-3’) based upon co-clustering of samples in multi-dimensional scaling plots. Cartilage from the group defined ‘Clinical Group 2’ shared an association with ‘Osteophytic’ samples, which both demonstrated relationships with the H2 and H4 module eigengenes. These two modules overlapped with the C4 and C5 consensus modules, respectively. Ostensibly normal articular cartilage (from adults undergoing total knee replacements or adolescents) had an inverse association with these modules; **b**, Matrix shows the degree of overlap between human network modules and consensus modules; the absolute number of genes is provided and this is represented in the vertical graduated colour bar. The consensus modules shared between the species are associated with the H1-H5 modules. Modules of unassigned genes (H12 and C6) are not shown.

**Supplementary Figure 5: Rat module-trait associations** – **a,** Module eigengenes were correlated with cartilage and chondrocyte phenotypes or traits including *in vivo* and *in vitro* studies. Matrix cells show correlation values (top line) and associated *p*-values (bottom line). Colour intensity (right side vertical bar) indicates the positive (red) or negative (blue) correlation between a trait and module eigengene. For example, chondrocytes in alginate beads (‘Alginate’) were strongly correlated with the R15 (black) module eigengene, whereas those from two-dimensional monolayer (‘Monolayer’) had a negative correlation with the same module. Samples from different ages (‘Neonate’, ‘Young’, ‘Adult’, ‘Aged’) demonstrated some distinct associations with module eigengenes; neonatal and adult/early aged samples demonstrated reciprocal associations with the R5 and R18 modules annotated for immune system process. Additionally, samples derived from different growth plate zones (‘Resting’, ‘Transitional’, ‘Hypertrophic’, ‘Proliferative’) were also found to exhibit distinct module associations. Samples from *in vivo* models of OA (‘Intervention.Group1’, ‘Intervention.Group2’) did not cluster into published sham or surgical interventions and were assigned to groups based upon co-clustering of samples in multi-dimensional scaling plots. Cartilage from the group defined ‘Intervention Group 2’ demonstrated relationships with a number of modules (R5, R8, R9, R11), which corresponded with the C4 and C5 consensus modules; **b**, Matrix shows the degree of overlap between rat network modules (R1-R19) and consensus modules (C1-C5); the absolute number of genes is provided and this is represented in the vertical graduated colour bar. The consensus modules shared between the species are associated with several rat modules including R1, R4, R5, R7-9 modules. Modules of unassigned genes (R20 and C6) are not shown.

**Supplementary Figure 6:** Network representations for rat and human modules that overlap with C4 consensus module. **a**: Network derived from highly connected nodes in both the R8 and R9 modules, which overlap with the C4 consensus module and are associated with cartilage from a subset of rat joint intervention studies. **b**: Network derived from highly connected nodes in the H4 module, which overlaps with the C4 consensus module and is associated with cartilage from a subset of human cartilage samples. In both networks *Ecmn* and *Kdr* are highly connected, but the degree (number of connections) differs between the rat (total of 69 connections for each gene) and the human (total of 10 connections for each gene); weaker connections have been removed for clarity. Both genes are defined as hubs with high *k_ME_* values (rat – *Ecmn* = 0.82, *Kdr* = 0.83; human - *ECMN* = 0.9, *KDR* = 0.85). Figure key describes the size of nodes and the thickness/colour of the edges.

**Supplementary Figure 7: Network for Rat R12 module**. **a**, The R12 modules is associated with sham samples (saline injections into joints) and annotated for genes related to skeletal system. Module contains classical cartilage-associated genes (*Col2a1*, *Acan*, *Comp*); the most highly connected genes in the module were *Slc38a3*, *Lms2*, *Ptch1*, and *Chst3*. The strongest weighted edge was between *Col9a1* and *Col9a3*. Only weighted edges >0.05 were chosen for clarity; node size varies with degree; edge thickness and colour varies with edge weight. An equivalent *COL2A1* containing module was absent from human co-expression networks; **b** and **c** – when the expression of *COL2A1*, collagen type II, alpha I chain, gene was assessed across whole cartilage samples from humans there was no evidence of differential expression by clinical groups (**b**) or age (**c**).

**Supplementary Figure 8: Defining age-associated genes in human cartilage samples**- **a**, genes from the R2 module (showing moderate association with aged rat cartilage samples) were used to develop a gene signature that would discriminate between young and old human cartilage (**Supplementary Data SD42**). A representative signature of three genes (*BCL6*, *ID2*, and *OMD*) separated healthy and OA cartilage by PCA, but not healthy samples from older individuals; **b** – box-and-whisker plots show differential expression of BCL6 between young and old cartilage samples (*p*=5.4e-3), but this was replicated between healthy and OA samples; **c** - In rat cartilage samples from different ages there was a reciprocal trend for Bcl6 expression (*p*=0.034).

**Tables**

**Supplementary Table 1**: **Functional annotation of modules arising from rat gene co-expression network analysis**. Each module is described by (column 1) the colour assignment and number of genes, (column 2) module preservation statistics for conservation of rat modules in the human network, and (column 3) gene ontology functional annotations for biological process (BP), metabolic function (MF), cellular compartment (CC), and KEGG canonical pathways where p<0.05 after Bonferroni correction. The most significant term for each analysis was used. Modules are presented in rank order based upon the module preservation scores (**Supplementary Data SD35**).

**Supplementary Table 2**: **Functional annotation of modules arising from human gene co-expression network analysis**. Each module is described by (column 1) the colour assignment and number of genes, (column 2) and (column 2) gene ontology functional annotations for biological process (BP), metabolic function (MF), cellular compartment (CC), and KEGG canonical pathways where *p*<0.05 after Bonferroni correction. The most significant term for each analysis was used. Grey modules are genes that were unassigned and are shown for clarity. Colours refer only to human modules and are not directly comparable to rat modules. Modules are presented in rank order based upon the module preservation scores (**Supplementary Data SD35**).
