# Supplementary material for: Cross-species gene modules emerge from a systems biology approach to osteoarthritis
Source: NPJ Syst Biol Appl. 2017 May 17;3:13. doi: 10.1038/s41540-017-0014-3 (PMC5460168; doi:10.1038/s41540-017-0014-3)
Supplement: Supplementary file 2 — Supplementary Material [file 41540_2017_14_MOESM2_ESM.zip › Supplementary_Material/Supplementary_Methods/Supplementary_Methods_Notes.docx]

**Cross-species gene modules emerge from a systems biology approach to osteoarthritis**

*Mueller, A.J.* ^a^*, Canty-Laird, E.G.* ^a,b^*, Clegg, P.D.* ^a,b^*, and Tew, S.R.* ^a,b,^***

^a^ Department of Musculoskeletal Biology, Institute of Ageing and Chronic Disease, Faculty of Health & Life Sciences, University of Liverpool, William Henry Duncan Building, 6 West Derby Street, Liverpool L7 8TX, United Kingdom

^b^ The MRC-Arthritis Research UK Centre for Integrated Research into Musculoskeletal Ageing (CIMA)

*Corresponding author: Dr. Simon Tew, Address - as above. Telephone: +44 (0)151 795 6235. Email:[tew@liverpool.ac.uk](mailto:tew@liverpool.ac.uk)

**Supplementary Methods 1:**

Notes to Users:

Example code and prepared data files are made available for the purpose of reproduction and to encourage future development of this work.

Data sources are provided in Supplementary Data SD 43 and 44. Some replicates were excluded from studies (these are defined by the accession codes), but this exclusion was performed after pre-processing and during the quality control of each individual study. The authors provide summarized .RData files upon which the further analysis was performed.

The codes provided (folders A to E) follow the workflow and analysis pipelines provided in Supplementary Figures 1-3. These codes do not cover all the available functions possible in WGCNA; excellent tutorials are provided by the developers^[[1]](#footnote-1)^. Additionally, the iterative assessment of the data and manipulation of parameters is not provided.

All WGCN analysis was performed using v1.49 or earlier. These earlier versions can be found in the CRAN repository. Changes to the blockwiseConsensusModules function in v1.51 should be noted when using later versions.

1. https://labs.genetics.ucla.edu/horvath/CoexpressionNetwork/Rpackages/WGCNA/ [↑](#footnote-ref-1)
